# Supplementary material for: Experience of healthcare personnel on Co-payment mechanism and the implications on its use in private drug outlets in Uganda
Source: PLoS One. 2024 May 17;19(5):e0297416. doi: 10.1371/journal.pone.0297416 (PMC11101086; doi:10.1371/journal.pone.0297416)
Supplement: S1 Appendix — (PDF) [file pone.0297416.s001.pdf]

Key Informant Interview Guide Pharmacist

1. Interview code ☐☐
2. Sex: Female ☐ Male ☐
3. Highest Health professional education level : B.Pharm ☐
   Dip. Pharm ☐
   MSc ☐
   PhD ☐
4. What is your position in this health facility……………………………………
5. How many years of experience do you have in your current position ……………………

OR

Introduce yourself …

Questions

1. What is copayment mechanism in malaria treatment (Probe: Green leaf ACTs, subsidized dispensing price of ACTs/Green leaf ACTs, QAACTs)
2. What antimalarial agents are stocked in this pharmacy (Probe: Antimalarial mix, determinants of which antimalarial to stock, stocking of 'Green leaf' ACTs/QAACTs)
3. How do patients/clients access ACTs in this pharmacy (Probe: prescription based, Over-the-counter, recommendation by the dispenser, what guides dispenser recommendation)
4. What are the ACT antimalarial purchasing behavior of clients/patients to the facility (Probe: Ability to afford full ACT dose, Inability to afford to pay for the full ACT dose, frequency of occurrence of the inability to afford the full ACT dose, how do you address the challenge of inability to afford full ACT dose among the clients/patients to the facility)
5. What are the prices of ACTs in your facility (Probe: Dispensing price for cheapest and most expensive ACT, determinants of dispensing price for the ACTs, relative dispensing cost/price of ACTs stocked in your facility)
6. What are the non-ACTs that patients access/purchase for malaria treatment in this facility (Probe: frequency of occurrence, which non-ACTs are requested by clients/patients, why do clients/patients request for these non-ACTs for malaria treatment, are they prescribed or are requested over-the-counter)
7. Any other issue regarding use of antimalarial agents in Uganda you would like to share

MAKERERE UNIVERSITY
SCHOOL OF BIOMEDICAL SCIENCES
APPROVED
VALID UNTIL
★ 26 JUL 2022 ★
RESEARCH & ETHICS COMMETTEE
P. O. BOX 7072, KAMPALA
