# Supplementary material for: Experience of healthcare personnel on Co-payment mechanism and the implications on its use in private drug outlets in Uganda
Source: PLoS One. 2024 May 17;19(5):e0297416. doi: 10.1371/journal.pone.0297416 (PMC11101086; doi:10.1371/journal.pone.0297416)
Supplement: S1 File — (PDF) [file pone.0297416.s002.pdf]

Participant_Apac district

**CATEGORY OF INTERVIEW**	KII_PHARMACIST
**ORGANIZATION**	DRUG SHOP
**TITLE**	PHARMACIST
**GENDER**	MALE
**DURATION OF INTERVIEW**	37:51

Interviewer: I have started the recording

Respondent: Respondent says his name

Interviewer: And what is your highest level of education?

Respondent: Msc

Interviewer: What is your position in this pharmacy?

Respondent: Manager

Interviewer: how many years?

Respondent: Its now 4 years

Interviewer: Thank you so much, ah, there is a practice called copayment, have you heard about copayment mechanism in malaria treatment?

Respondent: Copayment, hmm, (someone enters and disrupts)

Interviewer: Ah, thank you, that copayment mechanism, you have not heard about it?

Respondent: Not yet

Interviewer: Alright, in the ACTS that you have in this pharmacy, have you observed the primary packaging of those medicines very well?

Respondent: From the industry?

Interviewer: Yes

Respondent: Not yet

Interviewer: The packages that you give to the patients, do you observe the primary packaging, the boxes where the medicines is contained

Respondent: Yes

Interviewer: You see it very well?

Respondent: As they came in the cartoon, the package, yeah (confirming)

Interviewer: But even those that you pick to give to the patient, you also see them?

Respondent: Yes

Interviewer: Have you seen the color of the Greenleaf?

Respondent: Yes

Interviewer: On the medicines?

Respondent: Yes

Interviewer: Okay, do you understand what the Greenleaf signifies in that packaging?

Respondent: Yeah, ah, I still don't understand much but some bit of it

Interviewer: Okay, what is it?

Respondent: Its like, ah, Greenleaf, that coartem, that medicine for malaria, they are the best rather than the antimalarials which is coartem.

Interviewer: Okay, what makes them the best?

Respondent: Its like their reaction is faster, and then they are stronger than any other.

Interviewer: Hm, are you saying that for the ACTS, that you have here, the ones with the Greenleaf on them are the best?

Respondent: Yeah

Interviewer: Okay, ah, so you have those that are Greenleaf and those are not Greenleaf?

Respondent: Yes, we do have them

Interviewer: Is there difference in the pricing?

Respondent: No, not much difference in price but getting Greenleaf is a problem, Greenleaf, you can get them once in a while, sometimes it disappears but sometimes you get them without that Greenleaf on it.

Interviewer: Okay, so, also because the Greenleaf is difficult to get in the market, is that contributing to the prices at which you are dispensing?

Respondent: No, the price does not matter but where we can get it in bulk is a problem?

Interviewer: What, ah, who are your suppliers? For the Greenleaf coartem?

Respondent: Yeah, Greenleaf, actually is made by Sipler then we usually get it from distributors like ABACUS pharmacy. Those are the people we usually get from.

Interviewer: Sure, great, what antimalarials do you stock in this pharmacy?

Respondent: Actually, they are many

Interviewer: You could just brief me, you could just take me through the major ones.

Respondent: I can outline for you the major ones for ones,

Interviewer: Sure

Respondent: One we have coartem, it has three varieties of them, 4 one is coartem which is with 24 tablets, this one where you take 4 in the morning, 4 in the evening,

(Interviewer interrupts)

Interviewer: I understand what you are trying to say with coartem but I think you specific brands

Respondent: Yeah

Interviewer: Could you take me through the actual brands you have not the, (respondent interrupts)

Respondent: The actual brands

Interviewer: Yeah

Respondent: There is Lumartem, that is first, where you take 4, there is Lariact DS, DS means double strength, you swallow 2 morning, 2 in the evening. Then, there is ah, sorry I am forgetting the name. They are only 6 tablets but I am forgetting the name,

Interviewer: Its okay, we shall recover it.

Respondent: Its only 6 tablets where you swallow only one. Those are quadral (quadrat) strength. Then second one, we have lonart tablets, then we have Artesunate., D-Artepp

Interviewer: Are the tablets of Artesunate single medication or combination medicine?

Respondent: The double

Interviewer: Artesunate and what?

Respondent: And Amodiaquine, those ones you swallow 2 2 per day. Then D-Artepp, those you swallow 3, there is D-Artepp, there is P-Alaxin, there is duocotexin. But all of them they are of the same content but different brandings. You get P-Alaxin which is containing, (tries to remember name) this one here, I am forgetting again, it's a combination (there is some silence).

Interviewer: So you have listed, ah, ACTS, do you also have non-ACTS in stock?

Respondent: Yeah

Interviewer: Which ones do you know?

Respondent: The ones we have, there is, which one?

Interviewer: The antimalarials which are not ACTS

Respondent: Yeah

Interviewer: What else do you have?

Respondent: Ah, in fact, they are the ones I was telling you after the quardral (quadrat) strength. There is P-Alaxin.

Interviewer: But P-Alaxin is not.. (Respondent interrupts)

Respondent: Yeah, yeah, sorry, it contains that artemisinin (mummers), I have sigrim

Interviewer: Sigrim is what?

Respondent: I will show you the articels, we have antequine

Interviewer: Artequine is?

(there is silence)

Okay, you have artequine, what else?

Respondent: We have Fansidar

Interviewer: Sure

Respondent: We have quinin, we have chloroquine

Interviewer: Do patients come into the pharmacy ask for non-ACTS?

Respondent: Yes

Interviewer: Which ones do they normally ask for?

Respondent: Actually, I have some people, most of them, for those people who come specifically for what they want, like, ah, Fansidar, some ask, like, "I want Fansidar." There are those who use fansidar alone. Then, there are some few people who use chloroquine alone.

Interviewer: Sure, as a manager is this pharmacy, what determines the quantity and type of antimalarial you stock?

Respondent: Actually, the consumption, it is the one of them, what else determines.

Interviewer: So, consumption is one of them, what else determines?

Respondent: Then, other than consumption, we do ask the stock list we do experience, lack of capital, it also make us because sometime you stock any other drug apart from these antimalarials them the antimalarials will get finished before these other things get finished. So, it makes us get hard time in buying antimalarial again because the money was still in other drugs.

Interviewer: Do you have any considerations for Greenleaf ACTS when stocking?

Respondent: Yeah

Interviewer: What is that?

Respondent: One, we do consider the expiry dates, the expiry date, we want at least, if we are stocking, the expiry date which is nearer is 6 months, 6 months ahead because what we bring doesn't last for that duration but we need it at 6 months, that it can pass 6 months.

Interviewer: Alright, any other consideration?

Respondent: I think that's all

Interviewer: That's all?

Respondent: Yeah

Interviewer: Ah, how do patients access antimalarials in this pharmacy?

Respondent: They do access antimalarials in 2 ways; one of it is they do test themselves after of which they just come and say "this is form after I tested, I have used this drug and which one to use again?" so from here, we somewhere where you go and they prescribe for you then you come and buy. After prescribing for you, you come and we dispense for them. There are others with, let me say they know how they are feeling the they will just tell us this is, (leaves statement hanging), ah, what, malaria, then say give me antimalarials, or give me coartem or P-Alaxin, so we dispense to them.

Interviewer: So, you are saying, there are those who come with prescription and then there are those who come without prescription?!

Respondent: Yeah

Interviewer: When a patient comes with no prescription, what determines the medicine that you are going to give to them?

Respondent: Before we dispense to them, there is a question we always ask like "how are you feeling?"

Interviewer: And the person has already told you they have malaria and they want medicine, so what determines the kind of medicine you are going to give them?

Respondent: We first ask them what they have been using before.

Interviewer: Okay

Respondent: Which drug they have been using before and for how long they have taken of which you are feeling. After that we give them what fits them.

Interviewer: What do you mean by "what fits them?"

Respondent: The drug that they can swallow to reduce on the malaria parasite on them.

Interviewer: Okay, so, in terms of proportion, which of those types of access has the most number of clients? The ones that come with a prescription or that come with no prescription?

Respondent: Actually, they are almost the same.

Interviewer: Okay, so that is 50-50?!

Respondent: Yes

Interviewer: In terms of behavior of the clients in the pharmacy, purchasing behavior when they come to the pharmacy to buy these antimalarial, lets talk about the purchasing behavior of the clients that you get in this pharmacy. How do you look at their ability to purchase these medicines?

Respondent: Their ability to purchase, what I do see is most of them could come saying. This one I have been experiencing, lets say the disease they have been experiencing and they have been taking the drug, he comes back, so others can take like 2 dosages to wait for what, ah, any, lets say a family, any other person who will fall sick again. Yeah

Interviewer: Yeah, that is important, ah, we trying also to find out whether they can afford a full doze of say the medicine that they want.

Respondent: Yeah, 70% of the patients, they can afford a full doze.

Interviewer: Okay.

Respondent: But 25-30% can buy half, quarter a doze, half, quarter dose.

Interviewer: Okay, so, when someone is not able to buy a full dose, you give them half doze or quarter doze.

Respondent: Hm, (in agreement)

Interviewer: Okay, ah, what is (leaves statement hanging), you said that the frequency is 25-30%, so, when someone comes with 1000 Ug shs. You give them medicine for 1000.

Respondent: Yeah, that is 6 tablets of it.

Interviewer: Do you have any, ah, ways of addressing the issue of clients not able to afford full doses, the 25-30%? What do you do with them?

Respondent: Yeah, always I try to talk to them, advising them that taking, (leaves statement), I always advise them in terms of their capital. I advise that like "if you buy in bits like this, it is what, ah, expensive, secondly, if you buy in half like this, you will forget to swallow your drug in time, so what you do, you should be able to buy a dose.

Interviewer: So, you counsel them?

Respondent: Yes

Interviewer: Do you let them go without the medicine or you give them the medicine for the money they have?

Respondent: No, we don't let them go without the medicine because if they go without, then, that's another problem. So, we give them like a doze, and these people who are always buying half doze, half doze. They are people that are nearer here. They will be "budgetive" (meaning economical) by the time these ones they have swallowed, they have planned for the next doze. That's also their complaint.

Interviewer: Sure, so, in terms of the person, the patient has taken medicine for 1000, how many of those eventually complete buying medicine for the 3 days?

Respondent: Actually, I have people who can buy for 1000 now and come in the evening for the full doze. And other people could buy like 1000 now but the next 6, some people can forget now.

Interviewer: So, in terms of the proportion or percentage, these people who buy medicines in small quantities, 1000 shs, 500 shs, how many of them, in terms of percentages eventually complete the dose?

Respondent: In terms of percent, those who always complete their dose.

Interviewer: Because they buy in bits, how many of those eventually complete buying a full doze.

Respondent: That one, ah, in terms of proportion, 40% of them.

Interviewer: Like 40% of those who buy in bits eventually complete the dose

Respondent: Hm, (in agreement)

Interviewer: So, the other 60% don't come back

Respondent: They forget.

Interviewer: What makes them forget?

Respondent: One, what makes them forget most if they have taken, lets say the first day, the second day they will feel better and they will forget,

Interviewer: Alright, so that's okay, ah what are the dispensation prices for the antimalarials in this pharmacy? if you could take me through the different prices for the different brands of antimalarials.

Respondent: Yeah, ah, those that have 24 tablets, we just sell 4000 shs.

Interviewer: Which brand is that?

Respondent: That is Lumarten, with Greenleaf.

Interviewer: Lumarten with Greenleaf is 4000 shs.

Respondent: 4000 shs. Then, there is one with, Lariact, that is also 4000, one without Greenleaf is 3500 shs.

Interviewer: So, you are saying that the low Lumaterm which has a Greenleaf, you are selling at 4000, and then the other one are, (respondent interrupts)

Respondent: 3500

Both: And it is not Greenleaf,

Interviewer: So, you are selling Greenleaf at a higher price than the non-Greenleaf.

Respondent: Yes

Interviewer: Why?

Respondent: One, that one getting is hard and then also the price, there is a small difference in their prices even in purchasing.

Interviewer: So, you purchase coartem – Greenleaf more expensively than the non-Greenleaf.

Respondent: Yes

Interviewer: Ah, okay, alright, huh, other price?!

Respondent: Then, after, ah, that one, there is another coartem that has double strength, you swallow 2 in morning and 2 in the evening, that one is 6000 and then, there is this one I was telling you, artefern, 6 tablets of which you swallow one one, one in the morning, one -eveing, so that one is also7500 and then I have D-Artepp. D-Artepp is also 7000 also, then I have, ah, there is one thing I have jumped there, lonart, it is 4500, almost the same as coaterm, same tablets, same way of swallowing but different brands, hm.

Interviewer: Alright, which is the cheapest antimalarial in this pharmacy?

Respondent: Coartem

Interviewer: No, there are, okay, coartem is a brand!

Respondent: Yeah

Interviewer: Would you want to give the actual brand of the cheapest ACT?

Respondent: Yeah, it is called Lariact.

Interviewer: Is it green leaf or not Greenleaf?

Respondent: Not Greenleaf

Interviewer: Not greenleaf, so you are saying that the non-greenleaf ACTS is the cheapest?

Respondent: Yes

Interviewer: Which is the most expensive ACTS?

Respondent: The one which is the most expensive is Duocotexin

Interviewer: Duocotexin?!

Respondent: Yeah

Interviewer: And duocotexin, is it greenleaf or non-greenleaf?

Respondent: Non-Greenleaf

Interviewer: Huh!

Both: There is no Greenleaf.

Respondent: yeah

Interviewer: so, you have said the cheapest and the most expensive, so, what are those prices? What is the cheapest and the most expensive price?

Respondent: Lariact is 3500 and Ducotecin is 14000

Interviewer: I see, what determines the dispensing prices of these medicines?

Respondent: Okay, what determines a lot is one, demand, there are those with high demand like p-alaxin. Even when someone has tested 1+ of malaria, they want p-alaxin and they are used to it because they take it and get better.

Interviewer: In terms of prices, you have Greenleaf and non-greenleaf, and you are saying that the non-greenleaf is actually cheaper than the Greenleaf?

Respondent: Yes

Interviewer: How do these compare with the non-ACTS in terms of prices, which one is more expensive, the ACTS or the non-ACTS?

Respondent: The non-ACTS are more expensive

Interviewer: So you are saying that you sell chloroquine more expensively than the ACTS?

Respondent: Yes, they also come more expensively from the wholesalers

Interviewer: What is the price of chloroquine, full dose?

Respondent: That is 21000

Interviewer: How about fansidar?

Respondent: Single dose is 1500

Interviewer: So if you want to do for 3 days, you multiple by 3

Respondent: Yes

Interviewer: Meaning that the non-ACTs are more expensive?

Respondent: yes

Interviewer: Alright, why do you the clients ask for the non-ACTS when they come to the facilty?

Respondent: There are those who are used to them in that even when they take other drugs, they don't get well, and when they take the non-ACTS, they heal faster.

Interviewer: Which non-ACT is most requested by clients?

Respondent: Fansidar

Interviewer: And what are the others that follow?

Respondent: Quinine, then chloroquine

Interviewer: Do they come with prescriptions or without prescriptions?

Respondent: Small quantity of them come with prescription

Interviewer: Where do the prescriptions come from, main hospital or private clinics?

Respondent: Mainly private clinics and once in a while from the public hospital

Interviewer: They write that someone should get a non-ACT like chloroquine?

Respondent: Yes

Interviewer: And you receive these prescriptions?

Respondent: Once in a while we get

Interviewer: Okay, and for those without prescription, how do they determine the non-ACT that they need?

Respondent: Earlier before I was tell you that they are used to it, and when they are feeling some signs and symptoms like headaches, they just go back to the previous experience when they had malaria and from there, they will run straight to this

Interviewer: In terms of proportion, what proportion of clients that buy non-ACTS come with a prescription?

Respondent: It is very little, it is about 20%

Interviewer: So that means majority come with no prescription?

Respondent: Yes

Interviewer: If someone comes without a prescription and they want a non-ACT, What determine the non-ACT that they eventually go with?

Respondent: They don't come as if they want dosage, they will take, for-example 10 tablets of fansidar and when you inquire how they will take it, they say I am used to it, so in case of any attack, I will swallow it the same way I did before

Interviewer: As we conclude this interview, I want you to elaborate how you deal with the challenge of clients not being able to afford a full dose in this pharmacy. What measure do you have?

Respondent: As I talk, there is improvement. As I told you, I have now taken 4 years. Improvement is in such a way, most people who were coming with no prescription, they now come with prescription after they have tested and then come for the medicine

Interviewer: So, you say that there is improvement that they come with prescription, if a client comes with no prescription but wants antimalarial ACT, what do you do to address this?

Respondent: We have a nearby clinic, we don't assume if they accept to cooperate with us to test. Then there are those don't accept. If they accept, they test and come back with the right results, so we can give them the right drug.

Interviewer: Last, the inability to afford a full dose, what have you been doing?

Respondent: There is a designation of how they can it, one, we don't sell 1 or 2 tablets, we sell 6 tablets if you promise to come back for more. When you buy 6 tablets, you take 4 in the morning and the

remaining 2, you will have to come back and buy more 6 for evening and next morning. Before there were people who were buying only 2 tablets but now we no longer do that.

Interviewer: What made you stop selling 2 tablets?

Respondent: There were many cases where many people were worsening. So, for us to prevent that, we decided to sell 6 and with this, even if the condition wants to worsen, a dose has been taken. They assume 1 tablet is enough.

Interviewer: Do you have scenarios where a dispenser would recommend a certain antimalarial to a client?

Respondent: Yes

Interviewer: What determines as a dispenser would recommend to your client?

Respondent: After a client has told me what they are experiencing, the signs and symptoms of malaria, I will inquire for the drug they have been using and for how long they have taken it, if the time frame is close, I can give the second line treatment.

Interviewer: Thank you much, regarding what we have discussed so far, would you surmmarize or would you like to add any other thing regarding antimalarial drugs, their stocking and sale in this pharmacy?

Respondent: I want to first appreciate you for coming and doing this research because from this you will know what people really need, for-example we find difficulty in getting the Greenleaf drugs because of production and it is not in the market. So, find somewhere to fix this one so that we can antimalarial drugs with Greenleaf because the demand is high.

Interviewer: Thank you so much.

Participant_Apac district

**CATEGORY OF INTERVIEW**	KII_PHARMACIST
**ORGANIZATION**	DRUG SHOP
**TITLE**	PHARMACIST
**GENDER**	MALE
**DURATION OF INTERVIEW**	34:40 minutes

Interviewer: Starts the interview

Respondent: I am called mr. (Mentions name) and I am a pharmacist and I am the director of (mentions pharmacy name) and I have 4 years of work experience

Interviewer: Thank you so much for welcoming us in your facility, I will go on and ask you some few questions. I don't know if you are okay with it?!

Respondent: Yes, I am very okay with it

Interviewer: Okay, thank you so much

Interviewer: I will start with if you really know what copayment mechanism means in malaria treatment?

Respondent: No, I don't know what copayment means

Interviewer: thank you so much for that. Ah, what of the word ACTS. Have you ever heard of what it means?

Respondent: Yes I know what ACTS means.

Interviewer: What could it really mean?

Respondent: ACT means artemisinin combined therapy

Interviewer: And what do they normally mean by that?

Respondent: Okay. Ah, it means, in the simplest terms they are meaning combining antimalarials to get the biggest efficacy, combining more than 2 antimalarials from a palnt called artmether

Interviewer: Okay, which kind of antimalarial agents do you have in your facility?

Respondent: The common ones are artmether lumefantrine, the common brands are coartem, lumartem. Then we have p-alaxin, doucotexin, d-artepp.

Interviewer: Thank you, have you ever heard of the term antimalarial mix?

Respondent: Antimalarial mix! I think, not sure

Interviewer: Okay, what do you normally mix with antimalarial drugs for your patients?

Respondent: In treatment of malaria, people normally have fever, normally you give them antipyretics like paracetamol, and others also come with some infections so we give them amoxil.

Interviewer: What determines your stock?

Respondent: Basically in Apachi, we have a high prevalence of malaria and antimalarial drugs are one of the stock that I have in large quantities because it moves very fast.

Interviewer: What makes you purchase combait, lariact or coartem?

Respondent: Sometimes the cost, the population here is very poor, so we go for the artmether lumefantrine drugs and not those other expensive ones like duocotexin.

Interviewer: Thank you so much, after you have stocked your medicines, what do you normally do to ensure that their quality is maintained?

Respondent: Before we start a pharmacy, there is the national drug authority that come and ensure that your premises is suitable enough to carry out a retail pharmacy business, we ensure that every medicine is kept in the described storage conditions, temperatures below 25 degrees, we have fans, ceillings, the room is cool as you have seen.

Interviewer: Thank you so much. How do the clients access the antimalarial agents in this facility?

Respondent: We have two types of clients, there are those who come with prescription from the clinics and hospital, they come after they have done the test, you see the results clearly that it is +2 and coartem has been written down, so we give them basing on the prescription. Then there are patients who come with nothing and when you ask them if they have tested, they tell you that they tested while others tell you that they didn't have money to test. So, some come without the prescription and we have to give them over the counter.

Interviewer: What do you consider when dispensing to patients who don't come with a prescription?

Respondent: Normally we ask them some questions about the signs and symptoms that they are experiencing, if they have fever, vomiting. Most times they agree to having all those signs and symptoms, then we can dispense to them.

Interviewer: How many come with no prescriptions?

Respondent: They are not so many. Normally they come with prescriptions

Interviewer: Thank you so much. What is the ACT antimalarial purchasing behavior of clients in this facility?

Respondent: The challenge I have is you find an adult treating him/herself with antimalarial drugs but he comes with 1000 and needs 6 tablets because we have a pediatric dose of 6 tablets, you tell them that this is a pediatric dose, however, they insist to have it and promise to come and buy more tablets. That is the main challenge we face here. So, we end up giving them drugs over the counter. Some of them don't even come back, that means they end up with the pediatric dose because they felt better.

Interviewer: If you have that challenge, what do you do as a pharmacist to address it?

Respondent: Mainly we ask who the treatment is for, sometimes we insist so that they buy the full dose, but on humanitarian ground, you feel like they will come back. You can refuse and they walk into another pharmacy and buy from there, this is business you know. So, it is a very big challenge we have and I don't know how we are going to fight it because these are clients, you want to be nice to them. You educate them about these things but it is up to them but we try our best

Interviewer: In your own approximation, what is the percentage of those who can afford and those who cannot afford a full dose?

Respondent: The percentage is like 25%, it is not a lot but it can contribute a lot. It is about 25-30%

Interviewer: 25-30% are those who do what?

Respondent: Those who buy under dose. The rest can afford a full dose

Interviewer: Which information do you accompany these people who cannot afford the full dose?

Respondent: We always advise them to come and buy more tablets to complete the dose. If they cannot completely afford, the hospital is nearby, they can go there and get free drugs since it is a government facility at a free charge. The only challenge is they fear the number of people there and wasting time there because you can take an entire day waiting for coartem of the numbers. It is one kilometer away from here so it is a walkable distance.

Interviewer: Don't you think your facility is aiming to getting more clients as the numbers are at the hospital are many?

Respondent: That could be true, I cannot tell but they always come

Interviewer: So, did you look at that when setting up your pharmacy, as many people don't get medicine from the government facility?

Respondent: Not really. I opened it to serve the community, not because people are not getting medicines from the government hospital. Sometimes the hospital runs out on drugs, other times people come at night and the OPD is not open at the hospital, so we help them at night.

Interviewer: Thank you so much, let us look at the pricing at this facility. What are the prices of the ACTS?

Respondent: I have coartem 6 tablets, it does for 1000, coartem 12 is 2000, then coartem 24 is 3500

Interviewer: So which brands of coartem do you have?

Respondent: I have the coartem brand itself, then combiat, lumertam then lariact. All these brands go for the same price.

Interviewer: Don't you have any other ACT a part from coartem?

Respondent: No, it is basically that one

Interviewer: How much do sell P-Alaxin and others?

Respondent: P-Alaxin is 10000, Duocotexin is 15000. Those ones are a bit expensive

Interviewer: P-alaxin is an ACT or a non-ACT?

Respondent: It is an ACT

Interviewer: Thank you, are those the ACTS you have at hand?

Respondent: Yes, those are the ones that I have

Interviewer: Thank you so much. What is the cheapest ACT that you have?

Respondent: It is coartem, the 6 tablets at 1000

Interviewer: Is it a full dose?

Respondent: Yes it is a full dose for a child less than 15kgs

Interviewer: How about if it is an adult above 18 years?

Respondent: I give them 4 tablets 2 times a day, 12 hourly and that is 3500

Interviewer: Thank you, what is the most expensive ACT in your facility?

Respondent: It is duocotexin which is 15000

Interviewer: What determines these prices?

Respondent: We look at the market, we do a market survey and compare how others are fairing with prices, you shouldn't be far away from them. Now for coartem for adults, I sell it 3500 but most people sell it at 4000, P-Alaxin, most od sell it at 10000, for duocotexin, we sell it cheaper than Kampala pharmacies who sell it at 18000 yet we sell it at 15000.

Interviewer: So if it the market, what makes your prices extremely lower than those of kampala? Is it because you obtain these drugs at a cheaper price?

Respondent: Sometimes people over exaggerate prices if they want to cheap our poor patients. But 15000 is fair for both the client and I.

Interviewer: How do you deal with the competition that you have with other pharmacies?

Respondent: If your prices are very high you will not see customers, but for coartem you could get it at 2500 and sell it 3000 and get a profit of 500 but I have rent to pay and pay my worker.

Interviewer: Doesn't it put your business at a challenge that you have to address issues like rate and workers when you lower the prices so low?

Respondent: No I have not experienced anything like that because the turn is fine. I could get little but when the turn up is okay so I can cover up. Why would I sell expensively to get more profits yet I am getting 2 clients per day!

Interviewer: How many clients come for antimalarial drugs per day?

Respondent: I can estimate that they can be between 30 to 50 people for antimalarial drugs alone in a day

Interviewer: Why do you think that you have high numbers of people coming for the antimalarial drugs?

Respondent: I don't know if this place is an endemic region for malaria but I I think Apachi is an endemic area as you can see it covered up with swamps. I think that is the main reason, the swamp covers the entire place, you can see the town is really small because of the swamp. Even in the villages, there is a swamp. We have a lot of breeding areas for mosquitoes.

Interviewer: You know we have ACTS that have a Greenleaf and the non-greenleaf ACTS, do you know the difference between them?

Respondent: People want the Greenleaf ACTS and they always for them and when you tell them that they are actually the same, they don't believe you. But for Greenleaf, it was the government of Uganda that put it there. I cannot tell you much because I really don't know a lot about them but I know it was th government of Uganda that put it there.

Interviewer: So you don't know why they put there?

Respondent: Maybe to show the artmether plant since they are from that plant.

Interviewer: Are the Greenleaf ACTS bought at the same price as the non-greenleaf ACTS?

Respondent: I have not seen any difference in the prices, I order on a weekly basis, sometimes I receive Greenleaf, other times I get non-greenleaf. It may be all combiat, one having Greenleaf and another not having it but the prices are same and that is why our prices also remain the same

Interviewer: Let us look at the non ACTS. What do you understand by non-ACT?

Respondent: Those are antimalarials that are not combined to given us a better effect

Interviewer: Which non- ACTS do you have in your facility?

Respondent: We have quinine, fansidar and chloroquine, that is all

Interviewer: Among these ones, which is the most asked for by patients?

Respondent: Every patient that comes asks for coartem, few will ask for P-Alaxin and few will ask for duocotexin. The common people will come for coartem.

Interviewer: which non-ACT do people normally come for?

Respondent: Quinine tablets.

Interviewer: Why do you think they will come for it and leave coartem?

Respondent: Most times they tell you that they have been treating malaria and they were not responding so they are moving to the second line treatment. Then there are mothers who still believe that they should be taking coartem in their first trimester, they always ask for quinine. Then there are clients who believe that it is only quinine that works for them.

Interviewer: Do they normally come for prescription or over the counter?

Respondent: Quinine is always given on prescription expect for those who say they only use quinine, those ones always come for it without prescription. They claim that coartem doesn't cure them

Interviewer: When they come without prescription, do you give them the medicine or chase?

Respondent: We don't chase them away but as I said we inquire if they have treated it before, some say they had treated with coartem but didn't get any response, then you give them quinine, but ther are those who come from quinine straight away, we give them

Interviewer: Is there any other issue you may want to share with us regarding antimalarial agents in Uganda?

Respondent: People are really abusing coartem, we try as much to avoid it but it really moves and some people are not responding to them, they complete the dose but come back with the same signs and symptoms. I cannot tell if they really took the medicines or not because they are really many tablets and there are people who fear taking many tablets, maybe they only take for 2 days and leave the rest because they are feeling better and after a week they are back here. That is one thing we need to we need to educate people about. Then if they could reduce the number of tablets to like 2, there is a lot of pill burden. If we go back to the first question you asked me, maybe I know it, you could rephrase it because I didn't understand it, or you could tell me more about it

Interviewer: About copayment mechanism?

Respondent: Yes

Interviewer: We will look at it after

Respondent: Okay

Interviewer: Is there anything you may want to tell ministry of health about the affordability of drugs?

Respondent: Basically the ministry of health has done its best, they are providing coartem free of charge at the hospital but the challenge is the patient load is what is making patients to shy away from the hospital people go there in the morning and leave at 4pm after getting coartem and paracetamol alone. People see it as a waste of time, the best they can do is increase the number of facilities because the hospital is always over whelmed and the reason is malaria.

Interviewer: Thank you so much

Respondent: Lastly, the mian challenge is in the pharmacy or the OPD pharmacy, one reason is the government has not employed pharmacy personnels, you find that an entire district has one pharmacist, two pharmacy technicians who are dispensers but they have to see over 700 patients in a day. But the nurses are very many, they work from 8am to 2pm, a dispenser has to be there from 8am to 7pm every day, they don't have off-days. That is another problem, so patients will sit there at OPD till 4pm waiting

for coartem. So they next time someone will go back, they will be critically ill, otherwise they will come and buy corteam for 1000 and they take it, that is a pediatric dose, an adult has to take 24 tablets. So we need to increase the capacity. A pharmacy is very critical in the hospital but people undermine it, if someone doesn’t get medicine, it becomes a very big challenge, time wasting at the OPD pharmacy, and it is not because they are not working , it is because they are over whelmed by the patients. It one dispenser or two, they have to dispense, record, pack and call every patient. They should increase pharmacy personnel in the hospital setting and things will be okay. Someone will go at 9 and by 10am they will be leaving and that is very okay.

Interviewer: Thank you for sharing with us. These points that you have shared will reach the ministry of health and will be addressed.

Respondent: Thank you, I am grateful that you came to Apachi

Participant_Tororo district

**CATEGORY OF INTERVIEW**	KII_PHARMACIST
**ORGANIZATION**	DRUG SHOP
**TITLE**	PHARMACY DISPENSER
**GENDER**	FEMALE
**DURATION OF INTERVIEW**	30:04 minutes

Thank you so much, I am having an interview with a pharmacy dispenser in one of the pharmacies in Tororo district. The person is a female, certificate in nursing and currently works as a dispenser in this pharmacy. So, we are going to have a short discussion on arteminisin availability, stocking and pricing in this pharmacy. Have you heard of copayment mechanism?

Copayment mechanism?

Yes, in malaria treatment

No

Alright, how about Greenleaf ACTS?

I have

What have you heard about Greenleaf ACTS?

What I know is, Greenleaf ACT is, a slogan used to advertise antimalarials, so, in most cases when someone comes and says I want the ACTs, just know someone needs coartem.

Come again, you have said when someone says they want ACTS?

Yes, you just know they want coartem, to those people at least who have ever heard the advertisement of ACT, but mainly, most of them come and just say "I want coartem"

In your owning understanding, what do you think is the difference between a greeenleaf ACT and a non-greenleaf ACT?

The Greenleaf is mainly from the government and then these ones (meaning non –greenleaf) aren't.

What do you mean from the government? Are you saying the government supplies private pharmacies?

Not supplying the private pharmacies but they are authorized by the government to make them

So to get that point clear, you are saying the government authorizes the manufacture of the Greenleaf ACTS?

I think that is how I can understand it. That is why the advert is being run on

What do you see the difference between, if there is, between Greenleaf ACT and non-greenleaf ACT?

The difference in what?

Anything. Is there any difference between them?

Not much, I have seen that the Greenleaf ACTS are at least affordable, price-wise, they are affordable

So you are saying that their prices is a lower than the others?

Yes. But now when we talk about the others, the non-greenleaf ACTS, I think that's where I don't get the whole thing right, which ones are they? The lonarts, or? Which ones are they?

Any ACT that doesn't have a Greenleaf label in the packaging

Currently, we don't have any. I think I have not seen. I think that is why I am not answering you right. We mainly have the ACT with a Greenleaf.

Even lonart has a Greenleaf?

No, it doesn't

Do you have lonart here?

Yes we do have lonart

So lonart is a non-greenleaf ACT, alright?

It should be because it doesn't have a Greenleaf. So we go back to the prices are affordable.

So that is the major difference?

Yes

So, as a pharmacy, is there anything you do to ensure the quality of your medicines in the case of antimalarials?

Should I put it on the pricing? When you talk of quality, we have to look at the expiry date. Mainly it is the price and the expiry date

So those are the things you to ensure quality?

The way you receive your customers matters, when someone leaves, they believe they have taken the right thing, the quality, the way you handle customers, the person will leave convinced it is the right thing. Some of these things are not the best but the way you handle and talk to the patient, they will think it is quality.

Good, so, what antimalarials do you have in this pharmacy? If you could just mention

We have the Greenleaf, which is the ACT coartem, we have lonart, the one which is 24 and the one is packed 1*6, that is the double strength lonart, we have D-Artepp,

What is D-Artepp?

D-Artepp is also an antimalarial, it is actually under the same group as duocotecxin. So, we have D-Artepp, duocotexin, P-Alaxin, chloroquine, quinine tablets, fansidar, we have Aloquine, I think that is what I have in mind right now.

Alright, so you say the antimalarials that you have are Greenleaf?

We have Greenleaf also

What are the different brands of Greenleaf that you have?

We have only artefan

So you have never stocked any other Greenleaf other than artefan?

So far, that is what I have been seeing being brought

Only?

Yes

Why do you think they only stock artefan?

I think that is what is t their office, because we order for drugs and that is what is sent, because it is not us who buy, we order and they send.

Okay, thank you, so what do you determines the stocking of these antimalarials?

The market demand

Any other thing?

The quality of the drug, like how fast it reacts on the client, they could be a few that take a particular brand but when they take it, it acts well. So, demand and quality.

In terms of relation to other antimalarials that you stock, which one do you stock the most?

We stock the ACT, the Greenleaf, we cannot run with it.

So there has never been a time when there was no Greenleaf ACT?

That one moves both on whole sale and retail and it is the affordable drug, one may come and needs an antimalarial, actually most of them come without a prescription but because they have been using it, they come and say “I want coartem”, you ask why they want it “ I want it in the house to prevent malaria, or I I tested myself and I tested positive” so, we mainly stock the Greenleaf though there are others but that one is brought in large quantities

Thank you, and the Greenleaf that you stock is artfean?

That is what we have so far

Have you heard of any other brands of Greenleaf ACT in the market?

There was lumarten where I used to work from, they used to stock artemether.

So, in terms of the patients who come to the pharmacy to buy this medication, how do they access these medicines from you?

They buy them

How?

They come to the counter, either presenting a complaint, then of course you will ask some few questions and if they have taken the test, those who are willing, you tell them to first go and take the test and come back with a lab report, those who are willing will go, do that and come back with the result. Then those who are stubborn will say “I have come with my money and I want antimalarials” and it being business, you cannot chase someone away because you have dispense. You give but you advise accordingly as the person is taking the drugs.

So you say, sometimes they come and want the medicine to buy, I just wanted to pick your discussion on that particular point, how do they come? What do they use to enable you to give them the medicine that they want?

Like I said, someone will come with a complaint, presenting a complaint

How do you help them?

Of course you will listen to the complaint, then you will advise this person “according to this complaint, why don’t you go and carry out this test?” then after that, you can come out with the results.

How many of the patients that you have encountered, in terms of proportions, that you advise to go and do the test actually come back with the results?

Like in a day or week?

Any duration that is easy

In day, if 10 came presenting a complaint, I think 6 can accept, though sometimes, they may listen and don’t come back with results. Then after some time, you see someone and they tell you that they went and were given medication from there, then others can back with the results and present it.

So, when someone comes with the results and they present it to, do they have a prescription as well?

In most cases, a few come with a prescription when you send them like that, because they look at consultation fee. That is one of the challenges that I have seen, someone will go and come back and when you ask why they don’t have prescription, they will tell you that the consultation fee 20000 and someone cannot afford that. So they come with the results and tell you to help them after you have given the medicine, you tell them “take and after three days, come back and tell me how you are feeling” so with that I can as well get to know, in case of anything, I can then refer, you get to monitor them, so we always encourage them to come back.

How many actually come back?

They come back and tell you "last time you told me to come back but the other I couldn't come back but today I was passing by and I decided to come back, I took the drug and I got better and that is why I didn't come back." The ones that can come back in time, can come when someone has not improved, so you advice that he goes back to the hospital and see a doctor. Go to the main hospital where they will not charge you consultation fee.

So we move into something called the purchasing behavior, of course you have said some of the patients come with no prescription and some come without a diagnosis, so, for your case, when someone comes without a diagnosis and you have advised them to go and get a laboratory test as one of the options, what are the other options that you give?

Of course, you first find out the history of the patients, how long have you felt like this, (interviewer interrupts"

When everything has been done, you have told them "go and do a lab test" that is one option, I wanted to know, what are the other options rather than advising them to go and do a lab test?

I have already told you, So, which other option do you really think I can tell you?

So you are basically say, for these patients, there is only one option?

We tell them to go and carry out the test, actually, go and see a doctor, but if they cannot, (leaves statement hanging) others go and do the test and come back and say they want antimalarials that they have been using then you tell the patient "you will come back for review" that is how I understand it

Have you encountered patients who cannot buy a full dose of an act?

we don't encourage them here

But have you encountered them?

They re there, very many.

So you are saying there are very many patients who cannot afford a full dose?

It is not that they cannot afford, but they are just stubborn to buy a full dose. That is what we have seen. Someone will come and ask for what they will swallow for the day or tomorrow, we don't encourage that. So, the best we have done here at our pharmacy, if you are not taking a full dose, we don't give you.

So you are saying, in case a person is not able to afford a full dose, (respondent interrupts)

We don't give you and maybe what we can say, "being a pharmacy, we don't cut, maybe you can go to the drug shop in a village setting and they cut for you what you can afford but make sure you finish the dose, keep going there and buying the rest of the tablets remaining"

So how often do you encounter these people that are stubborn, those who cannot afford a full dose?

They are always there, almost on a daily basis, especially boda boda guys, they are very stubborn, but we don't encourage. One thing I have love here, most of them know that when they are coming to buy, they are taking a full dose, even when you are taking for a child, you take a full dose but you will give the

child little and rest of the balance, you preserve it for future use provided it doesn't exceed the expiry date.

So you have identified the issue of not wanting to buy a full dose, you say they are stubborn, are there those who are genuinely not able to afford?

No, at least with ACT, I have not seen that.

So it is just stubbornness but they have the money?

It is just stubbornness because when you insist, someone will be like "okay musawo, let me go and look around and come back" and then they come and buy.

So you are saying in this pharmacy you don't give half a dose?

We don't give half a dose.

So, if someone cannot afford, you let them go?

Yes

So, what are the prices of the antimalarials you listed above? Could you just highlight them and the dispensing prices of each?

It depends on how we are buying them of course, like lumaterm, we are selling at 3000 a dose,

Pardon, that is?

Lumatern, artfan full dose 3000

Is that adult dose or?

Adult dose.

How about pediatric?

We don't stock the ACT for the pediatric, so when you buy, we just advise you on how you can cut the adult dose to give to the child. So, we only have the adult dose which is 3000.

Okay, and is that the cheapest?

That is the cheapest because when you look at lonart, it is 5000, that is the one of 24 tablets, then there is for 10000, that is the double strength, then duocotexin which is 10000, P-Alaxin is 10000, D-Atepp is around 6000 -6500 and chloroquine, a few buy that now days but a sachet goes for 4000-5000, then quinine mainly goes on whole sale but a sachet goes for 2500 meaning a dose is around 7500 for an adult.

Okay, any second line ACT?

Artesunate

But artesunate is first line for severe malaria, how much do you sell it?

On retail, 4000

What strength?

60mg

So what would you say is the most expensive ACT in this pharmacy?

In this pharmacy, duocotexin

And what is the price?

It is 15000.

Okay.

Does redmol match? I don't think it matches

And the cheapest is?

The Greenleaf which is 3000

What determines these dispensing prices for the antimalarials in this pharmacy?

It is the buying price. It is the cost price. The purchasing price, let me out it that way

Any other determinant?

We are living in the world of competition, so we have to always find out what our competitors are selling then we put at the market price. The market price matters.

Okay, we have talked a lot about ACTS, can we talk about non-ACTS as well. What non- ACTS do you have in this facility?

Redmol, cyrium, those are the ones

Okay, how often do you encounter patients coming for non-ACT antimalarials?

They come with a prescription.

Like how often do patients come and they buy non-ACT?

Rarely, unless we do counter prescription ourselves.

So, in case patients take medicines that are non-ACTS, which ones are normally bought over the counter?

The ones we have, the ones I have told you.

What are those?

Redmol and cyrium

Okay, why do you think patients sometimes go for non-ACT antimalarials?

They don’t go for them, but of course, like I have said, it is a counter prescription and it is our duty to see that we sell these drugs, so, obviously, I will tell you we have this and that but you can try out this as well.

So, have you encountered a situation where a prescription has come and in that prescription there is a non-ACT antimalarial?

Rarely

If it happens, what antimalarial is it that they normally prescribed?

I think redmol comes rarely, because when these promoters come, we sent them to the private hospitals and clinics to promote their drugs, so, once in a while someone comes with that prescription.

Okay, so we are coming to the end of this discussion, lastly, we have talked about over-counter access of ACTs in this facility. Could you share your experience on this? Non-prescription access to ACTs in this facility. Talk about it. Does it occur occasionally and if it does, what do you think are the drivers of this practice?

I think it occurs often and to me I think people have gotten used to the coartem because on a daily basis, they buy it, we sell it in large quantities because as I said someone will come and say, “I want to put it at home as first aid drugs at home”. So they buy with or with no prescription but because they believe that “I have used this drug for so long and when I use, it makes me fine”

So, they say when they take it, it makes them fine?

Exactly, when you are used to a particular drug, you cannot change to another one, like you know if I have malaria, I will take coartem and be fine, so it is a psychological thing as they already have it that if I don’t take this, I will not be fine. So, it makes it the number one moving drug.

Sure, as we get to end of this discussion, are there any things in terms of challenges or enablers, lets start with enablers of ACT access to patients in this facility. What are the enablers? What do you think enables patients to access Greenleaf ACTS in this facility?

The price

And what are the things that inhibit or block?

In this particular pharmacy, what blocks them is that we don’t cut (meaning selling half doses) any drug. But for those who can afford 3000, they come and buy

Thank you for giving me time for this interview, I just wanted you to tell me any final things you would like to share given the objectives of this study, wanting to study the extent of adherence, the guidance of the ministry to make Greenleaf ACTS accessible to malaria patients. Is there anything you would ike to share with us in regards to stocking, availability of Greenleaf ACTS and making treatment accessible to the patients?

I think this goes to the ministry of health or the government, they should make sure that there is one drug that shouldn’t miss in the main hospitals so that everyone can be able to afford. Not only in the main hospital it also in these health centers, it should be a number 1 supplied drug either in the hospitals or health centers. Then, I would also request you people that once in a while, you hold

workshops for dispensers for such things so that we are enlightened on certain things. I think it is very important because we require knowledge on a daily basis, you have to keep on learning, so if we are given such opportunities, we can know how to handle certain things and how to go about it.

Actually, the ministry contracted us to conduct a study on the copayment system in the private facilities, my team was in northern Uganda and then the other terms are in the different parts of the country. Of course we couldn't pick all the service providers but this training has been done in the country and I think there should be more funding to do more of this training.

Do you know why I talked about that?

Yeah

Because if it was like a workshop, you don't get me to answer questions because I may not have answers in my head, but if we are like in a team, we go through different experiences, today I may be on duty, the other day my colleague is on duty, maybe she encounters some challenge, so maybe it is an opportunity to share out some of these things and to learn more, I think it is important once in a while if it is done like that.

Okay, lastly as we leave, I want you to tell me about the antimalarial mix in this pharmacy, are you happy with the mix in this pharmacy? Are there case when a patient comes and asks for a particular antimalarial and they don't manage to find it here and they have to go to another pharmacy?

It is rare, I personally I have not encountered that

Sure, every time a patient comes they always find what they are looking for?

Yes. We have

Alright, thank so much for this interview. It has been nice sharing your experience, we are now going to stop the recording of the interview

Thank you so much, thank you for the opportunity, thank you for coming because I have learnt 1 or 2 things also. Thank you so much, please come again

Thank you

PARTICIPANT _APAC DISTRICT

**CATEGORY OF INTERVIEW**	KII_PHARMACIST
**ORGANIZATION**	DRUG SHOP
**LEVEL OF EDUCATION**	BACHELOR IN NURSING
**TITLE**	DISPENSER
**GENDER**	MALE
**DURATION OF INTERVIEW**	34:46

Interviewer: Starts the interview by introducing himself

Respondent: (Mentions her name), I am the supervisor of this drug shop, I qualified in bachelor of science in nursing and I have work experience in the fields of about 37 years. After my bachelor, I was promoted as principal nursing officer of Lira regional referral hospital. I had a working experience there of 4-5 years and I retired, so, I am a retired nurse. So, I am here offering services to the local community in Apachi in form of a drug shop.

Interviewer: Okay, thank you so much miss (mentions respondent's name). it is a pleasure to be with you in this facility and thank you for accepting us to carry out this research study in your facility. I would wish you have some discussion with you, about the malaria and, my first question which I can start with is, do you have some ideas of what Greenleaf ACT means?

Respondent: Not quite, though I have heard about it though I don't have the idea about it and what it does exactly.

Interviewer: So, have you ever seen any other drug that has, which is maybe called a Greenleaf before?

Respondent: No, I have not seen yet.

Interviewer: Okay, should I see some of the antimalarials that you are having?

Respondent: Yes please

(they see the drugs)

Interviewer: I think we have seen them, ah, the drugs that you have. So, you have an idea of what Greenleaf mean?

Respondent: Yes, now I have. Greenleaf means, these are ACTS which have been subscribed (meaning subsidized) by the government making the prices a bit low otherwise they should have been very expensive.

Interviewer: Okay, thank you so much. What of the wprd ACT, have you ever heard of it? You have an idea of what it means and if you have an idea, could you please give us examples of it?

Respondent: ACTS are Artemisinin combinations treatment and they are those ones don't have (leaves statement hanging), these ones, the examples of ACTS which have greenleaf, I have methanine, that is Alefaine, then we have Lurmatem which is children's dose, then we have Artesunate combined with amodiumquin which is (leaves statement hanging), should I tell you the prices?

Interviewer: No, it is okay

Respondent: Examples of the Greenleaf, those are the only ones that we have, then examples of the ones without a Greenleaf, we have duocotexin and P-Alaxin.

Interviewer: Okay, thank you so much for that idea, let us go to what we call stocking of these ACTS that you have in this facility, you know when you have medicines, they don't come from heaven but you go and purchase them, not so, so e are going to look at your stocking of antimalarials, first, let us share which drugs you stock in your facility for malaria treatment?

Respondent: The drugs which I have for malaria treatment, being a new drug shop, I had thought of mainly oral so I have the artefane, lumartem, I have duocotexin, I have P-Alaxin . I avoided injectables because I was just a beginner, I didn't want tamper so much with those ones yet.

Interviewer: So, you have talked about the antimalarials you stock in your facility, which is very good, what really makes you on which antimalarial to buy for your patients?

Respondent: My motivating factor for stocking these very ones I have is because of the malaria which is very rampant in this district and it affects children, mostly women. So, I felt like let me get the first line treatment first instead of going for the second line because in case I encounter a patient who needed second line, that one would be beyond my drug shop. I would refer.

Interviewer: Is there any issue of pricing that you may say, "this drug I am going to get it because it is cheaper?" is there any other considerations that you put in your mind when you are bringing these drugs to the facility? Are there some more considerations?

Respondent: Yes, the first line treatment which I have, they are actually cheap and our local population here are actually poor, so that is why I have stocked this one which are a bit reasonable and effective. I considered its effectiveness and cheapness, so at least the population can afford.

Interviewer: You have talked about the Greenleaf antimalarials, if you go to purchase theses medicines, would you go for those with Greenleaf or any that you find there as long as it is for malaria?

Respondent: According to my observation, the Greenleaf are cheaper and effective.], so, that is why even us you see, they are more here, the other ones are just to keep other customers who need them.

Interviewer: Do you have any issue that someone would (leaves question hanging). Do you have patients who specifically want Greenleaf ACTS or you purchase it as you as a person because they are cheaper?

Respondent: First of all, because of my considerations and what I know about this population and the high rate prevalence of malaria over here, I went for Greenleaf ACTS because I knew it would be affordable, but then, some other customers come and ask for the ones which are expensive. That is why I stocked a few of the P-Alaxin and duocotexin.

Interviewer: And how often do patients come and say "I want Greenleaf"? Do you normally encounter that? Does it usually happen?

Respondent: Not quite, I don't want to deceive you. They don't ask for Greenleaf but they ask for coartem, which is of course the Greenleaf. That is the one they are familiar with.

Interviewer: Okay, thank you so much, still on the idea of stocking, to ensure quality of these antimalarial drugs while in your facility, what do you normally do because I am sure there are things you normally do to make your drugs safe for your clients, what are the things you do to ensure that the quality of these antimalarial drugs is good?

Respondent: Now, considering the quality and maintaining it, I have to stock my drugs in a safe place like in the cupboard, in the shelves and make they are not compromised by moisture and heat. And when I am working, I normally consider first in first out according to their expiry dates

Interviewer: Thank you for sharing with us about maintaining the quality of the antimalarial agents, and please do continuously maintain it, let us go to how patients come to your facility, how do patients usually access these antimalarial agents in your facility?

Respondent: Most of my patients come, usually to buy the drugs but I have some knowledge, I usually ask them "have you tested before" if they say they "I have not tested", I would request them if they are able to, let them first test and then we treat. But then, there are a few who will tell you " I had already test" then I supply them with the medicine

Interviewer: To the issue of testing, do you carry out testing in your facility?

Respondent: I do rapid diagnostic test at least to have me assess properly that this is malaria, otherwise the population would think every fever is malaria.

Interviewer: So, do patients come with prescriptions and how often do they come with prescriptions or most of them come and just buy the medicines over the counter?

Respondent: Few of them come with prescriptions, but most of them come just to buy over the counter

Interviewer: Most of them come to buy over the counter, what percentage? Could you approximate the number, like in a month I could have this number of patients come for medicine over the counter and which percentage do you approximate to be those ones who come with prescriptions?

Respondent: I would say, 30% would test, then about 30 come with prescriptions, then the rest come to buy over the counter.

Interviewer: For those who come to buy over the counter, is it a challenge to you? Or patients usually have in their mind that they want medicine? If patients come here and want to buy medicines without prescriptions, what do you do? Is there any other thing that you do to them?

Respondent: When they come to buy medicine directly, it is difficult to convience them, at times it is difficult, but most of the time when I talk to them, they accept to test and they do the test. It is really a challenge because, at times they resist, when you really insist, they say “I have already tested”. So, you find that it is really challenge, meaning, most of them take the drugs without being tested

Interviewer: For the patients who cannot afford the test, are there some signs and symptoms that you can look at and you say that this person is capable of getting antimalarials from my facility?

Respondent: Yeah, signs and symptoms of malaria are very clear, especially when the patient come complaining of headache, lack of appetite, chills, at times they even present with diarrhea, and then when you take the temperature, it is really hot, meaning above normal which is 37.8 or 38.

Interviewer: Okay, thank you so much, now let us look at the purchasing behavior of these medicines in your facility, what is their purchasing behavior?

Respondent: I have not got you very well

Interviewer: Okay, how do patients purchase these medicines, are they capable of purchasing a full dose or they cannot afford?

Respondent: Patients’ purchasing behavior is really challenging, as I told you before, this is a poor population, so they would prefer coming to buy for 500, meaning they cannot afford to buy a dose for one day. They will say, “let me first go and take this one and when I get small money, I will come and buy another one” and yet from my observation, some of them don’t come back to complete the dose, which means most of them who take the drug don’t complete the dose

Interviewer: So in your approximation, how many people can afford a full dose and how many people cannot afford a full dose treatment?

Respondent: Those who can afford a full dose treatment, the population is a bit low, may be 20% just, the rest, I just see them, they come and buy a few, some do come back but others don’t come back.

(seems to have pause the recording)

Interviewer: Pardon on the purchasing behavior

Respondent: As I was telling you, many come and want to buy the drug, other have tested before, other have not tested before and because as I told you the community is a bit poor, most of those who suffer from malaria cannot afford even this one (shows something), even a piece, they say “I will add”. You educate them that taking a less dose will make the malaria resistant, they say “I will add more” but most of the time, they don’t come back. But a few of them who have the money buy full doses, they go and take, I don’t see them coming back. But these other ones, they always come back, which means the malaria keeps on reoccurring

Interviewer: How often do the come and do that?

Respondent: I think in day, I see about 2 or 3 who come and cannot afford a full dose and they want to take pieces

Interviewer: As a supervisor, how do you address that challenge that someone cannot afford a full dose treatment?

Respondent: When somebody cannot afford a full dose, it is also a challenge to me because I don't take drugs on credit, so, I have just to educate them and tell them to come back for more, because if I keep on giving on credit, I might run out of drugs and I will run out of business.

Interviewer: Thank you so much, on the issue of the inability of these clients affording a full dose treatment, has it been a challenge to you?

Respondent: I feel it is a challenge and perhaps in the future, I would request, I don't know if it is possible for the government to subsidize more so that those who cannot afford will be able to afford because our main challenge in the community is, when they go to the hospital, NDS cannot also supply what is enough to meet the need for the community, that is why they come over to the drug shops. So, maybe I don't know if it is possible for government to support the drug shops somehow, so that we really assist this community

Interviewer: Okay, thank you so much. Let us look at the prices of these antimalarial agents that you have in your facility, could you please tell how much you are selling each and every antimalarial that you are having?

Respondent: I have coartem, I sell them at 3000, then lurmatem, which is half the dose at 1500, then I have duocotexin, those ones are a bit expensive at 15000, I have P-Alaxin at 10000, then I have artesunate, I sell artesunate at 2000 because I buy them at 1500, I just add 500.

Interviewer: Artesunate injection or?

Respondent: Tablet

Interviewer: Could you please show me that brand and I look at it?

Respondent: Okay

(they move and look at the brand)

Interviewer: Thank you so much for the prices, which one is the cheapest and which one is the most expensive?

Respondent: The cheapest here is artesunate and the most expensive (interviewer interrupts)

Interviewer: How much is it?

Respondent: It is 2000, I wanted to talk about the branding, lumartem is the cheapest at 1500, followed by artesunate which is 2000,

Interviewer: Then the most expensive one?

Respondent: The most expensive one and non-greenleaf is duocotexin at 15000

Interviewer: Thank you so much. How do you make these prices as you from the manufacturer?

Respondent: You are talking of manufacturer, how about the middle men?

Interviewer: Okay the wholesalers

Respondent: We get from the pharmacies in Lira. Ah, artefane, they sell to us at 2500, so we add 500 to make a profit. Actually most of them are in the range of just adding 500

Interviewer: What do you consider to add only 500 on those drugs?

Respondent: In business, there is a standard calculation of making a profit. Profit should be 28% of the cost and then you add transport, of course spreading it all through and then you calculate, then you get the range and then get a whole number. Meaning that if I calculated on 2500 that I bought, and then I calculated and got 150 (explains calculations)

Interviewer: Now there is that issue of changes in prices from the wholesalers, how do you address that issue? Do you any antimalarial that you have that keeps on fluctuating its prices?

Respondent: From my observation for the 4 months I have done this drug shop, the price for artemisnin base antimalarial, they are a bit stable, I don't if it is because they have been subsidized, but then these other ones are a bit fluctuating. One time you buy at 12000, another tome at 13000, so there is price fluctuation, I don't know who regulates them

Interviewer: And how do you address that challenge? Do you keep the price of that medicine at its standard that you have been selling it or you have a way that you deal with it?

Respondent: There is no way you can it constant, if you find price has gone down, we should also go down a bit. I told you that I am really for service provision really, not so much profit making, so I feel like if I have bought at a less price, when I come back, I also give it a fair price less than it was before. Then if I find it more, I shouldn't also run out of business, let it also be high a bit.

Interviewer: Thank you so much, let us go to the non-ACTS, could you please explain to me what non-ACT mean before we continue?

Respondent: Yes, the non-ACTs, we have chloroquine, quinine and fansidar. Those ones, they are not artemisinin based products of antimalarials

Interviewer: Do you have them in your facility? Which ones do you have at the moment in your facility?

Respondent: Unfortunately, I have not stocked any of those

Interviewer: Haven't you had patients come and ask for them?

Respondent: I have had patients come asking for them, but since the lockdown, I have not been able to move to the bigger pharmacies to make more purchase

Interviewer: So how do you address a challenge that a client will come for the non-ACT in your facility and you don't have it?

Respondent: Normally, I explain to them, "it is not here but I expect to bring it, next time you come back, you will find it" and indeed, I am working on it. We made some orders from pharmacies, they send their boys to ride on bodaboda (motorcycle) and bring our consignment if we pay for it. So I was also trying to look for money, then I make orders

Interviewer: Don't you find it a challenge that you are getting the medicine from a far place and how do you deal with it?

Respondent: It's a big challenge because it adds on the cost so much, but we have talked to our suppliers, we as the drug shop owners here, we make our orders and they bring it together. Otherwise on individual basis, it is very expensive

Interviewer: Do you have a challenge of getting these antimalarial agents from the hereby pharmacies around Apachi district?

Respondent: That is not a challenge at all but my observation is they sell the same price you would want to sell it which means you may not be able to sell. So if they are sell you 3000, then you come here and you sell at 4000, it is then a bit unbecoming because the population know the average, so it is a bit difficult getting it from within. It is better we get from the same source together, the other side.

Interviewer: For the non-ACTS that you don't have, if a patient comes with a prescription for the non-ACT, how do you deal with that challenge?

Respondent: That challenge is a bit easy because we have these big pharmacies which can meet their needs. So, when they come, I refer them to those pharmacies because you wouldn't want them to miss completely

Interviewer: Okay, for the time that you have been here, let me say that you have had these patients who are in need for these non-ACTS, have you come across some of them?

Respondent: Definitely, they have been coming and as I told you, I refer them to those bigger pharmacies to go and get their drugs

Interviewer: And how often have you seen them so far coming?

Respondent: It is not very frequent since they are also very expensive, we see like one in a week

Interviewer: For those who have come for them, do they come with prescription or they want them at hand, (meaning over the counter)?

Respondent: So far I have seen 1 or 2 with a prescription, but the rest come just to buy over the counter.

Interviewer: 1 or 2 in a month or year?

Respondent: The 1 or 2 I am talking about are in the 4 months that I have been operating

Interviewer: Thank you so much, is there any other issue regarding antimalarial agents that you would wish to share with us?

Respondent: Yes, now that I am aware of the reason these ACTs are cheaper, is because of government's subscription, I think they should not stop, let them continue. Being an endemic area, Apachi, if it becomes expensive, then we shall register more deaths from malaria, and if you went to the hospital, maybe in their registers, you would get more neonatal deaths which are of course brought by malaria and even malaria in pregnancy is very rampant here. So, I would wish, let them continue and let them not run out of stock.

Interviewer: Is there any challenge that you have experienced ever since you started selling these anti-malarial agents?

Respondent: Maybe the challenge I get is what we have already discussed before, that others are in need but cannot afford a full dose. That is the main challenge here, otherwise they do come for service, most of them may not afford a full dose and I am wondering how we are getting on, whether we are getting more resistance or not

Interviewer: Okay, how much is your test for malaria? And briefly tell us the means of testing you are using in this facility?

Respondent: The only test I do here is RDT and I charge it 2000

Interviewer: Okay, thank you so much. Have you ever heard of the term copayment mechanism?

Respondent: Pardon

Interviewer: Copayment mechanism

Respondent: Copayment?

Interviewer: Yes

Respondent: No

Interviewer: Thank you for sharing with us, I am so happy that you have shared with us about malaria, any other issue that you would want to share with us

Respondent: Not quite, but I am grateful for your time, coming around and also for the research you are doing, I know it will help this community, it will help the district, it will help the nation and also inform WHO about what is going on because I am aware that malaria was declared, Apachi was leading in malaria incidence so, when you complete these research successfully, the information will help us a lot. Thank

Interviewer: Thank you for sharing with us, have a good day

Respondent: Good day to you.

Participant_Kabale district

**LEVEL**	**Pharmacy**
**TITLE**	**DISPENSER**
**GENDER**	**FEMALE**
**Time**	**31 minutes**

Interviewer: I am (mentions name) a research assistant at Makerere University doing a research study on our topic which is Predictors of Local Emergence and the Spread of Artemisinin Resistance among Ugandan plasmodium falciparum parasites and today I am interviewing?

Respondent: I am (mentions name) a dispenser I did a diploma in pharmacy, and I have a seven-month experience at this pharmacy.

Interviewer: Thank you so much for allowing us to carry out this research study at your facility. Let's share something about antimalarial use in Uganda. I am going to ask you some few questions. Do you have an idea of what co-payment mechanism means in malaria treatment?

Respondent: I am not sure about it.

Interviewer: Thank you so much. Have you heard of an ACT? And if so, do you have some of the ACTs in your facility?

Respondent: Yes.

Interviewer: What do you understand by an ACT?

Respondent: It is the combined treatment for malaria.

Interviewer: Do you have examples of those drugs in this facility?

Respondent: We have Lonart, Duo-cotecxin, we have Lumartem, Fansidar, Artesunate injection. We also have quinine.

Interviewer: You said that the ACTs are combined therapies, which combined therapies are you talking about?

Respondent: The combined therapies we have are artemether-lumefantrine, we have piperaquine and dihydroartemisinin. They are just in different milligrams and different brands.

Interviewer: For the time that you have dispensed, have you come across ACTs with a green leaf? Or when someone talks about a green leaf ACT do you know what it means.

Respondent: No.

Interviewer: Have you seen any antimalarial pack with a green leaf on it?

Respondent: No, I am not sure.

Interviewer: You have said that you have never seen any green leaf on a pack of an antimalarial before?

Respondent: Yes.

Interviewer: Can you bring for me that artemether-lumefantrine and then we look at it. Can you see this green leaf?

Respondent: Yes.

Interviewer: Have you seen the green leaf?

Respondent: Yes.

Interviewer: Do you have an idea as to why the green leaf is present on that pack?

Respondent: Yes.

Interviewer: What do you think it represents?

Respondent: Artemether, because it is got from a plant called the artemether plant, and it has leaves.

Interviewer: How many of these with a green leaf do you have in your facility?

Respondent: It is one.

Interviewer: What is its name?

Respondent: Artemether-lumefantrine. Artemether is 20mg and Lumefantrine is 120mg for each brand.

Interviewer: Thank you so much for that. I think you have now seen a green leaf ACT. So, can you help me to list all the antimalarials that you have in your facility?

Respondent: In brands, we have Lonart, P-Alaxin. Lonart Forte. We have Lumether, we have Fansidar tablets, we have Duo-Cotecxin, we have Artesunate, that's what we have.

Interviewer: Is that all?

Respondent: Yes, we also have the artemether injection plus the quinine dihydrochloride injection too.

Interviewer: For the antimalarials that you have just listed for me at your facility, what guided you to make them available at this place?

Respondent: They are different brands, and they are of different strength and different dosages like a 4 x 2 for the elderly, however there are those like the Duo-cotecxin whereby someone takes one tablet twice instead of three times. Then for the injections for example, the artesunate, we know that in some severe conditions for example, in pregnant mothers in most cases we opt for the injections and then we have Fansidar because we use it so much in prophylaxis in pregnant mothers. Sometimes someone can tell you that they have been in a risky place for malaria and when they are back, they want to prophylax themselves and so we also use it in that scenario.

Interviewer: Thank you so much for that. Let's share something about accessibility. How do patients or clients access these antimalarials from your facility?

Respondent: Most of them come with prescriptions, that is, they have been tested and they are malaria positive, so I dispense. Then, there are also some cases when someone can come and only our pharmacy is open and the only one that they can access. In such situations, sometimes we test, but we only do it when we really have to help a patient in case, they cannot access a clinic. So, we test them and if they are positive with malaria, we treat them.

Interviewer: Let's look at those who come for the drugs over the counter, what if a patient cannot afford the testing fee and they are having malaria, what do you do for them?

Respondent: Since this is Kabale, I do not dispense an antimalarial when I have not tested because there are very rare cases that you will find someone positive with malaria. There

are some who will come and claim to have all the signs and symptoms of malaria or even say that they have been out of Kabale but if I have not proved that one is positive, I cannot give them the drugs because there are situations where one can come and convince you that they have malaria but then when you test, we find them negative. So, I cannot dispense an antimalarial if I have not tested a person or if they have not come with a prescription for that medication.

Interviewer: How would you address the challenge that a client doesn't have a prescription? Would you not dispense at all if they didn't have a prescription?

Respondent: I would advise them. Since there are few cases of malaria in Kabale, there are cases where one would come and think that they cannot get malaria, and when we perform the tests and assessment and find that it could be possible that the person has malaria. So, in such situations is when we find that we are forced to help the patient to do the test. And sometimes we can find them positive. But for a challenge where someone comes and wants the antimalarial but is not positive - at least for the patients that I have met, I counsel them and talk to them and get convinced because I give them examples of people who thought the same way they are thinking, and it wasn't so.

Interviewer: Thank you so much for that. What is the percentage of those who come with a prescription and that of those who do not come with a prescription per month?

Respondent: I think the number of people who come with prescriptions can be 2%.

Interviewer: What about those who come without a prescription?

Respondent: The ones who come without a prescription come with signs and symptoms and be positive. It may also be the same percentage because I can find that I've got two clients in a whole month whom I have treated with malaria and then I can get one or two whom I have tested and are positive.

Interviewer: Thank you so much for that. What is the purchasing behavior of the patients or clients for these antimalarial agents?

Respondent: Do you mean what they want most?

Interviewer: In terms of affordability, can they afford a full dose treatment or not?

Respondent: They can afford because Lumartem has always been Ush 4,000 and Ush 5,000 and that is always its range. So, I've not got a client that is surprised about the price so if anyone wants it, they can always afford it.

Interviewer: Have you ever encountered patients that cannot afford a full dose treatment?

Respondent: No, the only challenge that I have encountered is that we have adult doses for the adults and yet sometimes we get cases of kids whereby they have their own regimen and packaging strictly for the kids but for us we have medicine for adults apart from the artesunate injection which we have that is 60mg and the artemether injection of 30mg and the 120mg but as for tablets, since the cases are very rare and most of the cases we get are for adults we bring in the adult doses.

Interviewer: So, you haven't seen any patient who cannot afford a full dose?

Respondent: No, I have not seen.

Interviewer: We have talked about the purchasing behavior. Can you list for me all prices of the antimalarials that you shared with me?

Respondent: Let me begin with the cheapest. The cheapest which is the artemether and Lumefantrine 20mg and 120mg It is for Ush 4,000. Then we have the same strength of Lonart of 20mg and 120mg, we sell that at Ush 8,000. Then we have the P-Alaxin. It is at 40mg and 320 mg, we give it at Ush 12,000 to Ush 15,000. Then we have Duo-cotecxin where one takes three tablets only once, for this one a dose is at Ush 20,000. Then the Fansidar strip is at Ush 3,000 and then the quinine is for Ush 3,000. Then for the artesunate injection, we give an ampule of 60mg at Ush 5,000 and then the 120mg at Ush 8,000.

Interviewer: Any other, what of Lumether?

Respondent: Lumether is also Ush 4,000.

Interviewer: And Lonart Forte?

Respondent: It is also at 40mg and 240mg, we also give it at Ush 8,000.

Interviewer: So, in all these that you have shared, let's concentrate on the ACTs only. Which is the cheapest and most expensive ACT that you have in your facility?

Respondent: I will begin with the cheapest. The cheapest in brand is Lumether and Ipca which is Ush 4,000. Actually, the cheapest is quinine of Ush 3,000 followed by Lumether and Ipca at Ush 4,000. Then the most expensive is the duo-cotecxin of Ush 20,000.

Interviewer: What do you consider to sell the antimalarials at their price?

Respondent: I consider affordability of the drugs because there are some patients who come to request for Coartem at Ush 4,000. Then there are patients who do not like taking tablets and request for injections and the price doesn't matter to them.

Interviewer: Let's consider their pricing. For example, why would you sell at Duo-cotecxin at Ush 20,000 or Lumether at Ush 4,000?

Respondent: This is a pharmacy, so I consider my supplier's price. It will depend on how much that I am supplied that will dictate the price at which I will sell at.

Interviewer: Thank you so much for that. You have talked about ACTs. Have you ever heard of non-ACTs and if so, which examples do you have in your facility?

Respondent: I think the non-ACTs are the ones we had before we got Coartem in the market.

Interviewer: What does that mean?

Respondent: A non-ACT is one component of a drug in that medicine. For example, Ago-quinine it only has quinine as the active ingredient.

Interviewer: You have talked about quinine; how do patients or clients request for these non-ACTs in your facility? Do they come with prescriptions?

Respondent: The ones I give my quinine have prescriptions. However, there are patients who come and say that they have malaria but what works for them is quinine and of course, we shall give them what works for them.

Interviewer: How often do you see these patients coming for quinine?

Respondent: Not so much because we have a really low prevalence of malaria here in Kabale, so they are very few. The times I have dispensed quinine are three or four times only for the time I have been here.

Interviewer: Why do you think they come for quinine? Why do you think one may not come and take Artefan or Lonart?

Respondent: As I have told you, there are patients who come and say that quinine works for them.

Interviewer: And what happens to them if they take the ACT? Have you ever asked them why?

Respondent: The Bakiga are rigid and not every Mukiga wants to hear much from you or give you their time.

Interviewer: Is there any other issue regarding the use of antimalarial agents in Uganda that you may want to share with us?

Respondent: There is some issue that I see, there are some people who come and tell you that they have all the signs of malaria and even tell you that they know that they have malaria. Sometimes we ask them to take a test, but they refuse. So, I think what is bringing a lot of resistance is that sometimes they use the medicine unnecessarily when they do not malaria. Sometimes it could be a bacterial infection, or sometimes it could be a UTI and then someone would get the back pains, the joint pains the headache and then they would think it is malaria. Another thing is there are people who think that every fever is brought by malaria and that is so common in children. If a child has a fever, the parents will immediately diagnose that as malaria and then they would come and buy the antimalarials. And sometimes we do not take the time to ask for whom they are taking the medication or who prescribed for them. So, they do self-treatment without confirming whether they have malaria or not. It is not only here in Kabale, even in my district I see it. When they get signs and symptoms, they quickly run to buy Coartem and start doing self-treatment. That is why I think resistance of these drugs is so much because in the start it, was a good medicine.

Interviewer: Thank you so much. In general, how many patients do you receive buying an antimalarial in a month?

Respondent: For this one I will offer an average. I think like three. Leaving alone the ones who vie for drug shops. Last month I received three and this month I have so far received one.

Interviewer: Thank you for sharing with us about the antimalarials and thank you for participating in the research study that we are conducting.

Respondent: You're welcome.

Interviewer: All right. Have a good day.

Respondent: Likewise. I am (mentions name) dispenser.

Participant_Kabale district

**LEVEL**	**Pharmacy**
**TITLE**	**PHARMACIST**
**GENDER**	**MALE**
**Time**	**35 minutes**

Interviewer: I am (mentions name) a research assistant from Makerere University. We are still doing research on our topic which stands to be the Predictors of Local Emergence and the Spread of Artemisinin Resistance among Plasmodium Falciparum parasites. Today I am interviewing?

Respondent: I am (mentions name), I am a pharmacist and director at Palace Pharmacy Kabale. I have been working here for the last two years.

Interviewer: Thank you so much for accepting us to be in your facility to carry out this research study. Let's share something about antimalarials used in Uganda. I will start with a few questions. Do you have an idea of what co-payment mechanism means in malaria treatment?

Respondent: No, I do not.

Interviewer: Do you have an idea of what ACTs are, and what examples of ACTs do you have in your facility?

Respondent: I know what that means. It is artemisinin combination therapies, and we have some. We have artemether-lumefantrine and dihydroartemisinin. Those are the ones that we majorly stock.

Interviewer: Let's look at the brands of ACTs that you have.

Respondent: We have Artefan, duo-cotecxin and others. I may not have all of them in my head, but I can check all the shelves and tell.

Interviewer: Let's look at something called a green leaf ACT. Have you ever heard of a green leaf ACT before?

Respondent: No, not really. Maybe you could elaborate. I may know it from a different perspective.

Interviewer: Have you seen a label of a green leaf on any of the antimalarials that you have in your facility?

Respondent: No, I have not seen that.

Interviewer: Thank you so much for that. Let's look at all the antimalarials that you have stocked in your facility. Can you tell me all the ones that you have currently, both tablets and injections?

Respondent: We have artemether-lumefantrine combinations, we have dihydroartemisinin.

Interviewer: The brands.

Respondent: You want the brands?

Interviewer: Yes.

Respondent: Just like I said, I may not be knowing all the brands, but they are there on the shelves. We have Artefan, Duo-cotecxin, P-Alaxin. The others we have are SP sulfadoxine-pyrimethamine. And that is all that we have.

Interviewer: And what about injections?

Respondent: We do not have injections; we do not stock them because there are no clients or patients for them. We spend two or three months without anybody requesting for an antimalarial injection. So as a business we do not stock what does not move.

Interviewer: Okay thank you. What really guides your choice when stocking antimalarial agents?

Respondent: It is demand. As a community pharmacy we are driven by demand, that is, what patients want or what clinicians are prescribing is what we stock. So, even the antimalarials that we have are few as you may realize because we do not receive prescriptions for antimalarial drugs. And we are also driven by the quality of medicine. We stock the brand that we know has been working. That is what basically drives us, the demand and the quality.

Interviewer: Thank you so much. You have talked about quality. If you are really stocking quality medicines, how do you make sure that the quality of these

antimalarials is maintained in this facility?

Respondent: We buy from wholesalers that we value, or wholesalers that value quality. We stick to specific suppliers, and we maintain quality buy reading around or being informed of what is happening. Of course, even after buying them we maintain the quality by good storage practices. The temperatures and the humidity are monitored so that they do not deteriorate in quality.

Interviewer: Thank you for sharing in quality assurance. Let's share something on the accessibility. How do patients access antimalarials, mainly ACTs, in your facility?

Respondent: They access them by prescription and that is majorly how they do access the antimalarials. That is majorly how they access the antimalarials. But I can say, especially when I am around as a pharmacist, I occasionally give over the counter when I have done my assessment and I suspect an infection. I can give over the counter.

Interviewer: For those that you say you give over the counter, what do you consider when you are assessing the patient to rule out other infections and conclude that it is malaria?

Respondent: What I do is, I clerk the patient and by following the signs and symptoms, I can rule it out. We do a simple RDT test and then we get to know if it is positive or not.

Interviewer: What are the signs and symptoms that you consider to be able to give an antimalarial agent?

Respondent: Fever, when somebody comes complaining of fever, general body weakness, joint pains, vomiting or nausea and especially here in Kabale, if they have a history of being in a place which is prone to having a lot of malaria cases. For example, if they have visited the central or the north and when they come, they complain of those signs and symptoms. And as I have said, we do an emergency RRDT especially when we know it is going to take time to access a clinician. We are mandated by the law in the pharmacy and drugs act to offer emergency services in cases where the clinician is not present. So, we can do a quick RDT and give. When it is mild is when we give these oral ACTs, we give artemether-lumefantrine. We rarely give duo-cotecxin, after all, it is expensive. We rarely give that unless it is prescribed by a clinician. That is what I can say.

Interviewer: Thank you for explaining that. Let's look at the percentage of those who come with a prescription and those who come without a prescription per

month.

Respondent: Of the patients that we receive per month, 2% come with prescriptions. It is a very small number.

Interviewer: And those that obtain them over the counter?

Respondent: That one is like 1%. We rarely sell off those antimalarials.

Interviewer: Thank you so much for sharing on that. Let's detail the issue about the purchasing behavior of the clients for these antimalarial agents. Can they afford a full dose treatment or not?

Respondent The patients can afford and even if they cannot afford, we cannot give them less. Actually, some of them come and say that they cannot afford, and they ask us to cut for them a strip, but we do not give out strips. We give out packs of a full dose.

Interviewer: Have you encountered patients who cannot afford a full dose?

Respondent: Yeah, we have encountered them, and they usually ask us to cut for them a strip, but we do not. We tell them that we can only give out a full dose.

Interviewer: So, how do you address the challenge that someone cannot afford a full dose treatment?

Respondent: We refer them to government facilities where they can access these drugs free of charge. We tell them that if they cannot afford, they should visit a government facility where they can access antimalarials free of charge.

Interviewer: How often do you see patients complain that they cannot afford a full dose treatment?

Respondent: It is rare, but it has happened. Maybe one in ten can say they need some few of the tablets.

Interviewer: Thank you so much for that information. Let's look at the prices. Can you list for me the prices of all the antimalarials that you have in your facility?

Respondent: Artemether-lumefantrine combinations are given out at Ush 5,000 and dihydroartemisinin goes for Ush 15,000 and some brands like duo-cotecxin which is expensive can go for Ush 18,000 and then the SP goes for Ush 2,000 per dose.

Interviewer: Is that all?

Respondent: That is all that we have.

Interviewer: Which is the cheapest ACT and the most expensive ACT that you have at your facility?

Respondent: It is Artefan and the most expensive one is duo-cotecxin.

Interviewer: What guides you when pricing an antimalarial agent? What do you consider?

Respondent: We have a standard mark-up on every drug that we sell. So, the cost price determines the selling price after we have put a markup. So, even antimalarials fall under the category of all the other drugs that we sell.

Interviewer: Is it okay for you to share with me the standard markup that you use?

Respondent: The standard markup is 50%. We multiply the cost price by 1.5, but for those who cannot afford, we can discount it. We can discount to 1.4 or even to 1.3. So that would mean we can discount it to Ush 3,000 if it is possible.

Interviewer: Is there any other thing that you may want to share concerning the pricing of antimalarials in your facility?

Respondent: No, that is all.

Interviewer: We talked about the ACTs, let's also say something about the non-ACTs. What do you think they are, and what are the examples of the non-ACTs that you have in your facility?

Respondent: The non-ACTs are those that do not have the lumefantrine and the artemisinin. In our facility we do not have any but recently I was thinking of stocking quinine but because of quinine's side effects, it is unpopular, and it used to be very crucial or very useful in the first trimester in pregnant women, but it is no longer very popular after ACTs were given a green light to be used in pregnancy. So, because of the side effects, people no longer ask for them. In the past five months I have seen only one client who has come to ask for quinine. Also, the people who sell are supposed to write out of stock medicine depending on the demand for the community, but they do not write quinine. It is never on the shopping list always because the people don't use them. So, we do not have non-ACTs.

Interviewer: Have you seen patients coming for these non-ACTs before and if they have ever come, do they come with a prescription or not?

Respondent: I have told you that for the past five months I have seen one. And this was a health worker from a clinic, so he wanted us to supply them; and just a few, not a lot. Maybe he got a client who really needed a non-ACT, so he run to the pharmacy here to see if we have any and we told him we do not have.

Interviewer: In your experience, why would someone come for these non-ACTs and not the ACTs?

Respondent: I thought it was something to do with pregnancy because he wanted quinine. I didn't ask more but I suspect it was a case of first trimester pregnancy.

Interviewer: Thank you so much for sharing with us. Is there any other issue regarding the use of antimalarial agents in Uganda that you may want to share with me?

Respondent: For Uganda I may not have a general statement but for the area that I work in, the general statement is that we do not have challenges with ACTs. Prevalence is low and for some in the public institutions, we have antimalarials expiring. Even in public institutions like the hospitals, you would rarely get somebody looking for antimalarials. The people looking for them maybe have weakness in their bodies and they are just suspecting. So, generally I do not see resistance. I do not see it here in Southwestern Uganda. Because if it was there and it was a problem, I would've experienced it because I also work in public institutions. I would’ve experienced somebody not responding to artemisinin, but I do not hear that.

Interviewer: Have you not encountered any patient taking an ACT and it does not work for them?

Respondent: There is one I encountered some one and a half years ago. It was not here in Kabale, it was in Mbarara. I encountered that person because I was working in a community pharmacy there, and it was not working. He usually complained that the regular drugs that we used were not working on him. I do not remember which ACT he eventually used but he used one combination, but I do not remember it. You know, I want to concentrate in the cases that are common and read about the cases that are common. So, I do not make research on antimalarials because I do not see the challenge. But there is a person that was reporting that it was not working.

Interviewer: Thank you so much for sharing with me. Is there any word that you may want to share with us or advice that you can give us regarding antimalarials as far as the ministry of health is concerned?

Respondent: Of course, the advice is, in as far as the use of all medicines is concerned, we ought to ensure that all medicines are used rationally including antimalarials especially in areas that are endemic. They have to ensure that the drugs and medicines are used rationally and are obtained in the right way and that the dose is completed. Just like here, we know how amoxicillin is abused because cough is all over and people give it to themselves. Self-medication is high in amoxicillin, and we are experiencing a very high resistance. So, I tend to think that in areas where malaria is so high, they may also be irrationally using these ACTs. So, the government need to work very hard to make sure that we do not lose that drug and I tend to think and maybe as you do research you can also see how herbs, because people grow artemisinin. We used to grow artemisinin here in Kabale and people knew it was antimalarial and I actually think people in our community take it. So, they make a herbal concoction of artemisinin and take. If it is overused, can it not cause resistance in areas that you have observed resistance?

Interviewer: That is a good point.

Respondent: So, you try to look at whether the herbal concoctions that they use contain artemisinin and see whether maybe resistance is coming from that.

Interviewer: Thank you so much for sharing with us and for accepting us to carry out this research study in your facility. I thank you and have a good day.

Respondent: Thank you so much. It is a privilege. It was a good thing for me to participate in this. Thank you.

Interviewer: All right.

Participant_Kabale district

**LEVEL**	**PHARMACY**
**TITLE**	**DISPENSER**
**GENDER**	**FEMALE**
**Duration**	**35.15 minutes**

Interviewer: I am (mentions name) a research assistant at Makerere University. We are still doing our research on the topic which is Predictors of Local Emergence and Spread of Artemisinin Resistance among Ugandan plasmodium falciparum parasites, and I am interviewing?

Respondent: I have a diploma in Pharmacy I am working…, and I have three years of experience.

Interviewer: Thank you so much for accepting us to carry out a research study in your facility I want us to share something about the antimalarials that you have in your facility. Have you ever heard of co-payment mechanism in malaria treatment?

Respondent: No, I have not heard of it.

Interviewer: All right thank you. Have you ever heard of an ACT?

Respondent: Yes, I have ever heard of it.

Interviewer: Okay, can you explain more about what you understand about an ACT, and what are the examples of the ACTs you have in your facility?

Respondent: We have very many ACTs but the most common are Coartem, Lonart, duo-cotecxin and very many others.

Interviewer: Why do you think they are called ACTs?

Respondent: I do not know why they are called ACTs.

Interviewer: Have you ever heard of green leaf ACTs?

Respondent: Yes, I have heard of it.

Interviewer: How does it look like?

Respondent: These villagers of Kabale come and ask for a green leaf ACTs which they normally refer to as Coartem.

Interviewer: Do you have an idea as to why they are called green leaf ACTs?

Respondent: No, I do not know.

Interviewer: Have you ever seen a green leaf on any of the packs of the antimalarials?

Respondent: Yeah, I have ever seen it on this other Lumartem brand.

Interviewer: Why do think that mark is present on that antimalarial?

Respondent: I have not bothered to find out why.

Interviewer: All right thank you so much. Can you share with me how you ensure that the quality of these antimalarials is maintained in your facility?

Respondent: I make sure that I give the best quality. When someone comes and they do not have money I give them the cheap one but if one comes and I feel that they can take a good one I give them the quality drugs.

Interviewer: Okay, let's look at the ACTs that you have in your facility: how do you make sure that their quality is not distorted when they are present in your facility?

Respondent: I just make sure that I give them a correct dosage and I give them the directions and when I give out Coartem I tell them to take it with cold milk to make sure that it keeps good.

Interviewer: Thank you so much. Tell me all the antimalarials that you have stocked in your facility.

Respondent: I have duo-cotecxin. I have amodiaquine, I have Lonart DS I have this other ordinary Lonart. I have Combiart, Lumartem, and Artefan.

Interviewer: Thank you so much. What about the injectables; are there some injectables for antimalarials that you have in your facility? Can you share them?

Respondent: Yes, I have injectables as well. I have artemether injections and artesunate

injections plus quinine.

Interviewer: Share with me why you stock a given antimalarial agent. What's your choice?

Respondent: The demand forces me to stock certain antimalarials. I have customers in Ntungamo that prefer to use artesunate and artemether injections and that is why I stock it.

Interviewer: Thank you so much for sharing about the stocking. Let's share more about the accessibility. How do patients access these antimalarial agents in your facility?

Respondent: They are readily available so whenever they want the drugs, they find them here.

Interviewer: Do they come with prescriptions or most of them come and obtain them over the counter?

Respondent: Most of them come with tests and since I do not carry out tests here, they go to the hospital and test and when they come with positive results, I give them the antimalarials.

Interviewer: Don't you experience patients who come and obtain them over the counter?

Respondent: They do come. They come asking for antimalarials, so I make sure that I give them the best.

Interviewer: As you have said that you give them the best. What are the signs and symptoms that you normally look out for for you to be able to give out a given antimalarial agent?

Respondent: Malaria presents with a high fever, some sour tastes in the mouth, when someone is taking food, they do not have appetite. They are generally weak, and they have some joint pains. So, I consider those ones to be asymptomatic; they have malaria, but it is not showing, so I give them the antimalarials.

Interviewer: Let's look at the percentage of those who usually come with a prescription and the percentage of those who do not come with a prescription per month.

Respondent: The ones who come with a prescription are more than those who come without. But because we do not have many malaria cases in Kabale we get three who come without prescriptions in a month and seven who come with prescriptions in a month.

Interviewer: Thank you so much for that. How do you usually address a challenge when someone comes without prescription to obtain the medicine?

Respondent: If someone has no prescription sometimes it is very hard to diagnose them when there are some signs of malaria but when it is not malaria, there is the challenge.

Interviewer: Let's share something about the purchasing behavior of patients to these antimalarial agents: what is the purchasing behavior of patients or clients towards these antimalarial agents?

Respondent: The wholesalers have no problem, they come knowing what they want, and they buy in bulk but these other retailers who come with prescriptions normally want the cheap one which is Coartem.

Interviewer: How many clients can afford a full dose treatment and how many cannot afford a full dose treatment?

Respondent: Because we do not sell half doses, we encourage them to take a full dose and they normally do. For those who cannot afford treatment, I do not record them because I do not sell a half dose but for those who take a full dose, the percentage is very little because they are not many around here.

Interviewer: What is the approximate percentage?

Respondent: It is about 5%.

Interviewer: All right thank you. How do you address the challenge of clients who cannot afford a full dose treatment?

Respondent: For those who cannot afford the treatment, we just give them an analgesic like paracetamol and then we send them to the government hospital where they can get the treatment for free.

Interviewer: Thank you. How do you sell your antimalarials and ACTs? What are your prices?

Respondent: The cheapest one is Artefan, Lumartem, and Coartem. It is Ush 5,000.

Ordinary Lonart is Ush 10,000. Lonart DS is Ush 15,000, amodiaquine is Ush 15,000 and then duo-cotecxin is Ush 18,000.

Interviewer: What of the injections? What are their prices?

Respondent: Artemether injection is sold at Ush 6,000 for each injection and then Artesunate 60mg is Ush 9,000 and then 30mg is Ush 5,000.

Interviewer: All right thank you for sharing the prices. Which of the ACTs is the cheapest and which is the most expensive one?

Respondent: The cheapest is Coartem which is called Artefan and the most expensive is duo-cotecxin.

Interviewer: According to the prices, what do you look at to give a given antimalarial its price?

Respondent: I do not consider prices. I only consider what is good for the patient like if I give duo-cotecxin, that is good because it does not have pill burden like Coartem. With Coartem you take four pills in the morning and pills in the evening but for the others you take three pills once a day. That is what I normally consider.

Interviewer: You are selling Coartem at Ush 5,000 as you said. What really made you to sell it at Ush 5,000? What did you consider?

Respondent: I consider my cost price because I get it at a cheaper price than others.

Interviewer: Let's look at the ACT with a green leaf on it and an ACT without a green leaf on it. Are they of the same price?

Respondent: I consider them to be of the same price, but these patients prefer the one with a green leaf on them.

Interviewer: Why do you think they usually want the one with a green leaf on it?

Respondent: I did not bother finding out.

Interviewer: Thank you so much for that. Let's look at the non-ACTs. Which non-ACTs do you have in your facility?

Respondent: I do not think I have them.

Interviewer: Okay let's look at if you have quinine, Fansidar. Do you have that medicine?

Respondent: Quinine and Fansidar are there but they are not commonly used.

Interviewer: How often do you see a patient coming for Fansidar or quinine or chloroquine?

Respondent: They do not normally come. We give those ones to wholesalers. We do not have retailers for those drugs.

Interviewer: Okay thank you so much for that. Is there any other issue that you may want to share with us concerning the use of antimalarial agents in your facility?

Respondent: No. Not really.

Interviewer: Okay. Thank you so much for sharing with us.

Respondent: You're most welcome.

Interviewer: Likewise.

Participant_Mbarara district

**LEVEL**	**PHARMACY**
**TITLE**	**PHARMACIST**
**GENDER**	**MALE**
**DURATION**	**30.25 minutes**

Interviewer: My name (mentions name), a research assistant from Makerere University. We are doing a research study on our topic which stands to be Predictors of Local Emergence and the Spread of artemisinin resistance among Ugandan plasmodium falciparum parasites. And I am interviewing?

Respondent: You're interviewing (mentions name), I am a pharmacist. I have been in service for one year and I am happy to join you in this study.

Interviewer: Thank you so much for accepting us to be in your facility. I have something I want us to share. Have you ever heard of co-payment method in malaria treatment?

Respondent: No, it is my first time to hear of that. Maybe you can elaborate.

Interviewer: Have you heard of an ACT?

Respondent: Yes, I have heard of that.

Interviewer: Can you tell me what you know about ACTs?

Respondent: ACT is Artemisinin-combination therapy. This is what is used for the treatment of malaria, and it is approved by the NDA as one of the drugs used in antimalarial therapy. ACTs are a combination of other drugs for example artemether-lumefantrine which is branded as Coartem. And it has shown positive outcomes. Only that these days with the active use we have got response from our clients coming for second doses after finishing the first dose and this has prompted us to use third-line drugs where we have example of duo-cotecxin which is a second-line in this case. We also have

had artemether injectables at response from our clients, even after administering injectables they still come with the same physical presentations.

Interviewer: Have you ever heard of green leaf ACTs and non-green leaf ACTs?

Respondent: I am not sure of that question. Most ACT drugs come with a green leaf label. That is what I can say. I am not really informed about that.

Interviewer: Can you tell us some of the antimalarials you have seen with that label?

Respondent: I have seen Artefan, I have seen Coartem. I have seen artemether.

Interviewer: Thank you so much for that. In general, which antimalarials can you list for me that you have stocked in this facility?

Respondent: According to brands or generics?

Interviewer: Brands.

Respondent: We have Fansidar which consists of two drugs. We have Malanil which also has some derivatives in it. We have ACTs, we have Artefan, we have Coartem. Those are the two brands under ACTs then we have chloroquine, we have artequin. Amodiaquin. Then we have ACTs containing the dyhydroartemisinin which is duo-cotecxin.

Interviewer: Thank you so much for that. What is the determinant for you to stock these antimalarial agents that you listed for us in your facility?

Respondent: Actually, according to the antimalarial stock levels, we have a low stock of antimalarials but one of the reasons that makes us to stock antimalarials is because there is a necessity for each region or level to have antimalarials. We also based on reference treatments. I also stock because of marketeer influence. Then also we have to familiarize ourselves with the P. falciparum drugs. Such antimalarials are also P. falciparum drugs. So, we couldn't neglect the antimalarial stock.

Interviewer: What of the injectables for malaria. Do you have some injectables in your facility and can you give us some of them?

Respondent: We have quinine injections. We have artesunate. We have artemether.

Interviewer: Thank you so much for helping us know the stock that you have. For the stock that you have, what do you do to ensure that the quality of these medicines is maintained in your facility? What is the quality control of these antimalarials in

this facility?

Respondent: The quality control starts right away from the distributor. We ensure that quality is maintained right from the distributor and from the industry as a finished product. Before we receive them, we ensure that they are registered in a goods received book. In the process of registering, we check the physical appearance to ensure that the goods are not damaged and ensure that the secondary and primary packaging is not damaged and intact. Then we check the expiry date so that we do not receive medicine that is already expired or that the expiry date is not short dated. All those aspects are registered in a goods received note. We also ensure its traceability. Another thing is we maintain good quality storage of our products. As you can see our products are kept in clean shelves and they are not infested with pests, and we also ensure that they are kept under people aspect. And periodic checking is done when we are doing stock-taking. For antimalarial drugs that are in powder form like artesunate powder, we make sure that it is kept in a shelf, and it won't be allowed to get close to moisture or water. Also, not in direct sunlight. We have had cases of returned drugs and we encourage our clients not to use drugs that they suspect to have damages or maybe if there are some unused drugs, we tell them not to use them and take them back to the pharmacy. I think it is one of the ways to ensure rational drug use. There are some that are brought back to ensure that they are safely kept in their shelves because they are not being used.

Interviewer: Thank you so much for the information that you have shared in stocking. How do patients access these antimalarials from this facility?

Respondent: One of the ways is we dispense these drugs on prescription order. This is where a patient first goes through a diagnosis from a physician and then after a positive detection of malaria, they are written a prescription for antimalarials. I think it is a good thing because you at least know what you're treating. Most of the patients that have come here without prescription have an aspect that all fevers are caused by malaria. So, we encourage them to first go under diagnosis. One of the ways we dispense is by prescription and then another way is blind therapy. This is where a patient comes and orders for antimalarials on suspicion of malaria because of the clinical presentation or when a dispenser in a pharmacy will dispense a drug depending on the clinical presentation.

Interviewer: Looking at blind therapy, what do you normally consider giving it out? What do you look for in a patient to give a given antimalarial agent?

Respondent: One of the things that we do in this pharmacy is we have treatment policies

for different indications, and it is done according to the reference of some reference books following their treatment guidelines. For example, in this facility we use UCG which is one of the treatment guidelines for our country and we tend to benchmark on the clinical presentations on the indication. So, a patient is asked how they are feeling and in that way we able to take the patient's medical history and under all that process is where we manage to build a treatment policy for these patients.

Interviewer: Thank you so much for that. When we leave accessibility of these medications to these patients, let's look at the purchasing behavior of the clients. What is the purchasing behavior of these clients for antimalarial agents? I mean, are they able to afford a full dose? Can you talk about that?

Respondent: The purchasing behavior in this region according to dose as you have said, has been a positive one. Because most of the patients in this region, before buying drugs, the patient knows how it is taken and how long it will be taken. They even know the number of tablets in a full dose. Most of them will buy a full dose. I will put it at 99% that will buy a full dose. What I can say is that others tend to buy more than the dose. They can buy 2 or 3 doses such that if the first one has failed to work, they can go for the second one. Even if he has been advised that one dose is enough, they will go on to expand the doses given.

Interviewer: Thank you so much. You said that 99% can afford a full dose treatment. Let's look at the 1% that cannot afford the full dose. How would you address the challenge if a client comes, and they cannot afford a full dose treatment? How would you address this challenge?

Respondent: When it comes to that percentage of people that cannot afford a full dose, they come with their reasons. Most of them tend to say that they forgot their drugs somewhere or lost them. That is when we experience such cases.

Interviewer: I am looking at a patient who comes and cannot afford a full dose treatment and is present at your facility. Is this person going to be chased away or are they going to be given something?

Respondent: I haven't faced such a scenario but if it happens one of the things we do is to sensitize them on the advantages of finishing up the dose and the disadvantages of not finishing up the dose. If at all the patient does not have whatever it takes to finish up the dose, we divide the dose according to the days until they finish up the dose.

Interviewer: In terms of frequency, how often have you seen patients in a month or

a year who cannot afford a full dose and what are the reasons the give for not affording a full dose treatment?

Respondent: We often get very few patients on ACTs or antimalarials but a person who comes without enough funds to purchase the antimalarials often have a tendency of deceiving. Most times they say the drugs are expensive or they do not know what they are taking the drugs for. Because they have a bit of a fever, they tend to think that they have malaria. So, they think if they take two or three tablets of Coartem they may get relieved. That is the challenge we have.

Interviewer: Thank you so much. Let's look at the prices of the antimalarials that are present at your facility. Can you share the prices of each and every antimalarial that you have?

Respondent: Most of the common ones are the ACTs like Coartem or Artefan which consists of 24 tablets which goes for Ush 4,000/= to Ush 5,000/=. And the syrup goes for Ush 8,000/= to Ush 10,000/= and then the duo-cotecxin goes for Ush 15,000/= to Ush 20,000/= And then Fansidar goes for Ush 2,500/= to Ush 3,000/=. Chloroquine tablets go for Ush 200/=

Interviewer: What about the prices of the injectables that you have?

Respondent: Artesunate 60mg goes for Ush 6,000/= and the 120mg is Ush 8,000/= to Ush 10,000/=

Interviewer: For the antimalarials that you have talked about which is the least and most expensive antimalarial that you have?

Respondent: Let's start with Fansidar, Fansidar is the cheapest followed by ACTs for example, Chloroquine is the cheapest. For ACTs we have Lumartem which is Ush 4,000/=. The most expensive one is Lonart which is Ush 8,000/=

Interviewer: Thank you so much. For the ACTs that have a green leaf on their pack and the ones that do not have a green leaf on their pack, do you sell them at the same or different prices?

Respondent: Those ones with a green leaf are a bit cheaper compared to the ones without a green leaf. The price difference is about Ush 2,000/=

Interviewer: So, do you sell them at the same price?

Respondent: No, we do not.

Interviewer: Do you know what that picture of the green leaf means?

Respondent: I have seen it, but I haven't ventured to know why it has it. But all I know is that it contains artemisinin or that it is a green plant.

Interviewer: For the antimalarials that you have listed the prices, what guides you to choose the prices of these medications?

Respondent: There are a number of factors based on to determine the prices of the drugs, first of all we determine the distribution price or at what cost we receive the drug and what was the cost of transportation and as a health facility here we also have a business perspective. We know how much the profit margin is. So according to those three factors, we first revise them before we decide our selling price.

Interviewer: As you have talked about the ACT, I think you have heard of a non-ACT. Can you give me some of the differences and examples in each case of the two?

Respondent: ACTs are artemisinin derivatives and non-ACTs are not artemisinin derivatives. Examples of non-ACTs are quinine, chloroquine, amodiaquine and we also have Fansidar. Those are the ones I have.

Interviewer: How often does a patient come for non-ACTs.

Respondent: If it is not prescribed the patient will not come for them because they do not know them. Patients are more informed of ACTs.

Interviewer: So, they do not usually come for them?

Respondent: They rarely come for them.

Interviewer: Have you ever seen any in a month or in a year?

Respondent: If it is not prescribed a patient will not come for them. But in prescriptions we have seen some. In a year we may have like three prescriptions.

Interviewer: Thank you so much. You have said that patients do not usually come for them but why do you think they would come for the non-ACTs either with a prescription or not?

Respondent: It comes with patients' familiarization with these drugs. And most of the ACTs have been highly promoted through advertisement. Most of the ACTs have been advertised and patents are familiar with them. Not only with the patients but even with

the health practitioners they are all familiar with ACTs. So, most times if someone gets sick and they are prescribed for a drug it is ACTs that are given first.

Interviewer: Thank you so much. Is there any other issue regarding antimalarial agents that you may want to share with me?

Respondent: I think as this facility we have had cases where a patient is prescribed for ACTs, takes the first dose and gets no change and then comes for a second dose and still gets no improvement. So, I think we are also worried about the efficacy of the ACTs, and I think it would be useful if the public is sensitized about the use of these drugs because we've had many cases where patients come without prescription forms and many cases of self-medication. And I don't think it is of help when someone is treating themselves which has led to mass treatment failure with ACTs. So, I think the public must be notified and sensitized on how the drugs should be used.

Interviewer: Thank you so much for sharing with us. Any word for me?

Respondent: I think many treatment policies or programs have been enacted and have not been implemented. You've heard of programs like MDAT, MSAT MDA MFAT I do not know if you are familiar with them. They have been enacted but have not been implemented. If they are being implemented, I think we shall see a drastic fall in malaria prevalence in this region and a country. Why I think we are taking this study I am happy to go through it and participated. Thank you.

Interviewer: All right. Thank you too.

Participant_Mbarara

**LEVEL**	**PHARMACY**
**TITLE**	**DISPENSER**
**GENDER**	**MALE**
**Time**	**34.2 minutes**

Interviewer: I am (mentions name), a research assistant at Makerere University. We are doing a research study on our topic which stands to be Predictors of Local Emergence and Spread of artemisinin Resistance among Ugandan plasmodium falciparum parasites, and I am interviewing?

Respondent: (mentions name), I am a dispenser here I have two years of experience and I am a diploma holder.

Interviewer: Thank you so much for inviting us to your facility let's share something about artemisinin resistance and I will start by asking you if you have an idea of what co-payment mechanism means in malaria treatment?

Respondent: Surely, no.

Interviewer: Okay. Have you ever heard of what is called the green leaf ACT?

Respondent: Yes, I have heard of it.

Interviewer: How would you identify a green leaf ACT from a non-green leaf ACT?

Respondent: A green leaf ACT has a logo of a green leaf on its package.

Interviewer: Do you know what that green thing is?

Respondent: I think it indicates that that very drug was made from artemisinin. It is what I can think.

Interviewer: Why do you think that there are those with a green leaf and why there are those without a green leaf?

Respondent: Those ones with a green leaf have a combination of artemisinin in it and

those ones without a green leaf do not have an artemisinin combination in it.

Interviewer: If we talk about the ACT itself what does it mean, and can you give us some of the examples of the ACTs that you know or that you have in your pharmacy?

Respondent: First, ACT means artemisinin-based combined therapy and examples are Coartem and Lumefantrine then those ones which are non-ACTs that do not have the other artemisinin combination in them, and examples can be Fansidar, amodiaquine.

Interviewer: Could you give me some of the examples of the ACT brands that you have stocked in your facility?

Respondent: We have Lumertam, Comether and Artemether.

Interviewer: Thank you so much. When we look at the ACTs, how do they differ from the non-ACTs?

Respondent: As I said earlier, the ACTs have an element in them called artemisinin that the non-ACTs do not have.

Interviewer: Thank you so much. Let's look at quality assurance. What do you do to ensure that the quality of the ACT antimalarials that you have stocked in your facility is maintained?

Respondent: We keep them safe in a cool dry place. That is what I can say.

Interviewer: What else do you do to ensure that the quality does not deteriorate for these antimalarials that you have in this facility, is there any other thing that you do?

Respondent: Surely, nothing.

Interviewer: Thank you so much for that. Let's share something about the stocking of the antimalarials that you have. Can you tell me all the antimalarials that you have stocked in your facility?

Respondent: Coartem, Fansidar, Duo-cotecxin, Lonart, Artesunate injection, Quinine. Those are the ones I can remember.

Interviewer: When you talk about duo-cotecxin, where does it belong, is it an ACT or a non-ACT?

Respondent: I think Duo-cotecxin is a non-ACT. It does not have the green leaf logo.

Interviewer: Thank you so much for that. When we look at stocking, you have a variety of the antimalarials in your facility. What do you consider or what is the determinant for you to stock a given antimalarial agent? What do you look at to bring this antimalarial agent here at the facility?

Respondent: We consider a drug that is effective and what many people ask for.

Interviewer: Is that all?

Respondent: Also, these drugs that can work on many varieties of parasites.

Interviewer: Thank you so much for sharing with us about stocking. Is there any other agent that you have stocked that is not an ACT or any antimalarial that you use in the treatment of malaria?

Respondent: For sure, no.

Interviewer: Thank you for sharing with us about the stocking. Let's look at the accessibility. How do patients access antimalarial agents from your facility?

Respondent: Some of them just come and ask that they need antimalarials and we give them. Some of them come with positive results of malaria cases and we therefore prescribe for them.

Interviewer: For those who just come for the antimalarials without prescription, what do you consider to be able to give the antimalarial agents to them?

Respondent: I look for the weight of the patient and I find out the right dosage to give to the patient according to the weight of the patient and the age.

Interviewer: Are there some signs and symptoms that you look at and give the antimalarial agents to those that come without a prescription?

Respondent: There are some signs and symptoms of which they are headache, fever, chills, vomiting, and general body weakness. So, when we get a client with those signs we hire someone or we send them to a lab at health center and they get tested and then they give us the results and then we give them the right treatment.

Interviewer: Let's look at the possibility that a client or a patient cannot afford the

testing price or the money for testing. What do you do? Are you going to chase them away? What do you do?

Respondent: We do not chase them away; we do what they request us to do which is for us to give them the drug and then they go.

Interviewer: Thank you so much. Let's share more about the accessibility of these antimalarial agents. How often do you see patients coming without a prescription for antimalarials in this facility?

Respondent: Those people who come without prescriptions are much more than those who come with prescriptions.

Interviewer: How many do you receive in a month without a prescription?

Respondent: For those ones without prescriptions, in a month we can get like 20 or 30. But for a case with those with prescriptions we can get like five. They are very few.

Interviewer: Thank you so much for that. Let's look at something new which is purchasing behavior. What is the purchasing behavior of patients or clients for these antimalarial agents in your facility?

Respondent: I have not understood the question.

Interviewer: Can patients afford a full dose treatment and how many can or cannot afford a full dose treatment?

Respondent: Mostly, people come and tend to go with half dosages because they tend to complain that the drug is expensive and so they ask to take half a dose and then promise to come back later but then they do not come back.

Interviewer: So how many can afford a full dose treatment?

Respondent: I cannot tell the number but those who afford a full dose are somehow more compared to those who cannot afford a full dose.

Interviewer: Let's look the percentage per month.

Respondent: Those ones who cannot afford can be like 20 percent and the ones who afford can pay but with struggle.

Interviewer: Thank you so much for that. How do you address the challenge that a patient cannot afford a full dose treatment? How do you deal with it?

Respondent: First. When I see that a patient is totally defeated and is not having money. If I have something in my pocket, I can pay for him or her but if I do not have the money the patient will go away without anything.

Interviewer: Is that the way how you can deal with it?

Respondent: No, because it is not my burden.

Interviewer: Okay. Thank you so much. Let's share something about the pricing of antimalarials. Before we go any further can you give me prices of every antimalarial you have stocked in your facility.

Respondent: Yes, I can. Coartem at wholesale price can go for Ush 3,000/= and then for retail at Ush 5,000/=. Then a single dose of Fansidar goes for Ush 2,000/= and then duo-cotecxin goes for Ush 20,000/=. Lonart goes for Ush 8,000/= and then the artesunate injection 120mg can go for Ush 10,000/= and then 60mg of artemether injection can go for Ush 7,000/= and then 30mg can go for Ush 6,000/=.

Interviewer: Thank you so much for the pricing. Let's look at what you depend on to give these antimalarials their prices. In other words what makes you sell Coartem at Ush 5,000/= what do you do and what are the considerations.

Respondent: First, the first step is with the amount at which we bought it at. When we buy them expensively, we also end up giving a higher price.

Interviewer: Any other?

Respondent: That is it.

Interviewer: So, you consider the cost price only?

Respondent: Yes.

Interviewer: Thank you so much for that. For the prices that you have given us, which one is the most expensive antimalarial and which is the cheapest antimalarial that you have?

Respondent: The cheapest antimalarial is Fansidar which is at Ush 2,000/= for a single dose and maybe the expensive one in tablets can be duo-cotecxin which is Ush 20,000/= But when it comes to injections because a person takes a lot of ampules to complete the dose, artesunate can be much more expensive compared to tablets.

Interviewer: What of if we talk about the ACTs, which one is the cheapest and

which is the most expensive?

Respondent: I think ACTs are not expensive, like Coartem that can go for Ush 5,000/=.

Interviewer: So is it the cheapest?

Respondent: It is not the cheapest. It is the one with the least amount of money, but it is not easy for a client to raise.

Interviewer: And then the expensive one?

Respondent: The expensive one may be duo-cotecxin.

Interviewer: Let's look at the prices of ACTs with a green leaf and the ACTs without a green leaf. Are they of the same price?

Respondent: They are not of the same price. To me those with a green leaf are somehow cheaper compared to those without a green leaf.

Interviewer: Why do you think those ones with a green leaf are cheaper than those without a green leaf?

Respondent: I surely do not know the reason.

Interviewer: Thank you so much for that. Could you remind me about the non-ACTs that you have stocked in your facility?

Respondent: As I said, one was Fansidar, Amodiaquine.

Interviewer: How often have you seen patients coming for these non-ACTs in your facility? How often have you seen them?

Respondent: They come but at a slow rate. Patients mostly come looking for ACTs and not non-ACTs.

Interviewer: In your opinion, why do you think patients that come for these non-ACTs are very few?

Respondent: From my point of view, the non-ACTs are not common so most people may not know about them. But when you give an example of Coartem, it is known country wide.

Interviewer: Thank you. Let's look at how they obtain these medicines from the facility. Do they normally come with a prescription or not to obtain these non-

ACTs?

Respondent: Some come with prescriptions while others do not come with them.

Interviewer: What is the proportion or percentage of those who come with prescriptions?

Respondent: Those ones who come with prescriptions are very few for non-ACTs

Interviewer: How many approximately?

Respondent: In a month we can get like 10% of people who are looking for ACTs without a prescription.

Interviewer: Thank you so much. Is there any other issue regarding antimalarial agents in Uganda that you may want to share with us?

Respondent: First, it is about the expensiveness of drugs. Drugs have become so expensive to the extent that people cannot afford them. I do not know whether it is the issue of taxing. I cannot guess the reason but drugs have become expensive and yet many people are sick.

Interviewer: Is that all?

Respondent: That is enough for me.

Interviewer: All right. Thank you so much for sharing with us. Are there any other words you may want to share with us about antimalarials?

Respondent: What I can say is that if they can get a way of reducing the costs of antimalarials so that it can be somehow cheaper for those who do not have money.

Interviewer: All right. Thank you so much for inviting us in your facility.

Respondent: You're welcome.

Participant_Mbarara

**LEVEL**	**PHARMACY**
**TITLE**	**PHARMACIST**
**GENDER**	**FEMALE**
**Time**	**38.5 minutes**

Interviewer: You're welcome for this interview. Tell me about yourself.

Respondent: I am a pharmacist (mentions name of drug outlet).

Interviewer: That is in Mbarara district?

Respondent: Yes.

Interviewer: I am (mentions name), We'll be having a short interaction in the use of and access of antimalarial medicines in this facility. What is your position in this facility?

Respondent: I am a manager and the head of the retail business.

Interviewer: How many years have you spent here?

Respondent: One and a half.

Interviewer: Thank you so much. As I said, I am (mentions name) from the College of Health Sciences Makerere University and I am conducting a study in antimalarial access and use in the four districts of Apac, Tororo, Mbarara, and Kabale district. We are going to have a short interaction about this. As we start this discussion have you heard about the co-payment mechanism?

Respondent: No.

Interviewer: Okay, that is quite interesting there. In your supervision and day-to-day work in this pharmacy have you noticed that there are some ACTs that have a green leaf on them?

Respondent: Yes.

Interviewer: What do you make of that green leaf on the ACTs' packaging?

Respondent: I really haven't taken a keen interest in the green leaf packaging. Enlighten me on that.

Interviewer: In the pharmacy here; since you have not taken a keen interest in the green leaf, do you realize that there are green leaf ACTs?

Respondent: Yes.

Interviewer: In terms of how you dispense them regarding pricing, do they have the same price as those that do not have a green leaf on them?

Respondent: Well, the ones with a green leaf seem to be cheaper because I have a few brands that have a green leaf on them. On is an African brand, it has a green leaf, and it is a bit cheaper. But the rest seem to be at higher prices than the others.

Interviewer: Have you tried to find out why?

Respondent: I really haven't paid attention to that.

Interviewer: Okay. That means that you also buy them at different prices.

Respondent: Yes, we get them at a lesser price and the cost prices are lesser than the selling prices.

Interviewer: I don't know whether you have a keen interest in establishing the difference in the prices. What would be your analysis of the price difference between the green leaf and the non-green leaf when you are buying them as a pharmacy from the wholesalers.

Respondent: Of course, as I said, the green leaf ones' prices are a bit low and the ones without a green leaf are a bit higher.

Interviewer: All right. There is also another concept called the quality assured ACTs. When you are dispensing these ACTs to the patient do you ever think about the quality of the medicine you are dispensing to the patients?

Respondent: Yes, we do. When I think about quality, I want to serve something that I know will work for the patients. If I am not thinking about quality as a dispenser, I am doing a disservice to the patient. So quality is on top of our list.

Interviewer: That's good for the patients here. How do you ensure that?

Respondent: We make sure that we buy our drugs from licensed sellers.

Interviewer: Other than buying from licensed sellers, any other thing you do or that you consider?

Respondent: Of course, the drug has to be registered and the drug has to make the list of things we check to see on the quality list like the packaging, the batch numbers, the expiry dates. We verify our items and make sure that they should match. If there is an issue we have, we contact the seller.

Interviewer: Are you aware of anything from the Ministry of Health or what the malaria control division has specifically done in trying to ensure the quality of ACTs in the market?

Respondent: No. Not at all.

Interviewer: That is interesting. The Ministry of Health and the government are really doing what they can to ensure that patients get quality-assured ACTs. So, in terms of the antimalaria mix in this pharmacy, can you share with me the different antimalarials you have in this facility?

Respondent: Yes. Do you want the artemisinin-based only or all antimalarials?

Interviewer: All antimalarials.

Respondent: I have an artemisinin combination which is under ACTs. There is dihydroartemisinin and lumefantrine. Then I have Fancidar, pyrimethamine. I have Artequin which is an artemisinin combination. I also have Malanil.

Interviewer: Okay, it seems you have both ACTs and non-ACTs.

Respondent: Yes.

Interviewer: What determines your stock of antimalarials in this facility?

Respondent: It depends on the demand. Usually, first-line drugs are more in demand than second-line drugs and that is how we dispense our antimalarials. Usually, we do not dispense without tests. So, when a patient comes in and they think they have malaria and need an antimalarial what we do is we ask the patient to go and do a test and bring for us the results and we analyze them. We tell them to do an RDT and a BS and we see the results for if it is a plus 1, plus 2, or plus 3. So, we do not just dispense the antimalarials without a test.

Interviewer: So, the determinant for the stocking of the antimalarials is how fast they move? The demand?

Respondent: Yes. The demand.

Interviewer: Any other thing that you consider, or which influences your stock?

Respondent: Of course, even the price of the item because we do not want something which is very expensive. We want something that is of good quality and also not very expensive.

Interviewer: So how do you balance that?

Respondent: Of course, there are some companies that we know produce good quality medicine, but we also look out for the prices if they are not so high.

Interviewer: Okay. In terms of the clients in the pharmacy who come to buy these medicines how would you describe their purchasing behavior?

Respondent: Well. First of all, we do not have very many malaria patients that are over the record. So we have really few malaria patients. And of course, when someone has tested, and the test is positive, they try their best to get the medicine because I explain as a dispenser that if they do not do the treatment, it may progress to something worse. So we have to explain it and sometimes influence the purchasing habits of patients. Someone can say they aren't feeling that weak and that they can go without it. I'll have to explain to them what could happen if they do not take the medicine. So in some way we influence their purchasing behavior.

Interviewer: So by influencing you're saying that you make them buy medicine?

Respondent: In case the test is positive I can influence the patient to take the medicine.

Interviewer: Have you encountered some scenarios where some patients either do not want or cannot afford it.

Respondent: We have options that are a bit affordable. Prices range from USh 5,000. So, if someone cannot afford for example a double string tablet, we have an offer of a cheaper option which has many pills. One can take many pills in the morning and pills in the evening, and they do take and it still serves the same purpose.

Interviewer: So, in terms of this that you have just described. Have you come across someone who even when you have given them a cheap option still can’t afford?

Respondent: No. Not for the time I have been here. And we do not cut the doses, so the person has to buy the full dose. We tell them they have to take the full dose and we explain why they need to take the full dose.

Interviewer: So, whenever you find clients who want to buy the ACT. Do they all come with prescriptions?

Respondent: No. Not all the time. As I said, when someone does not come with a prescription and they have not done a test, I encourage them to go and do a test and when they have done a test, I can tell that the malaria is positive. And if it is, for example, a plus 1, I can give first-line drugs and if it is a plus 2 or plus 3, I will know what to give them.

Interviewer: In terms of proportions of the patients that you receive daily or whenever you get them, what would be the proportion of those that come without prescription and of those that come with prescription?

Respondent: The proportion I would say is 7/10. Although they are not that many. But the proportion is 7/10.

Interviewer: And none of them is not able to afford a full dose? Are all of them able to afford a full dose?

Respondent: Yes. Because as I said, we have options that are fit for anyone to buy. Honestly, I have not seen someone who cannot afford a USh 5,000 option. Maybe it is because of the location and the people coming. Maybe it is the class of people who come in here. But I have not seen someone who cannot afford it here.

Interviewer: Okay so in terms of the patients who do not have prescriptions, you have said that you request them to go and do the tests. But for those who still do not do the tests and suspect that they have malaria, how do you recommend the medicines for them?

Respondent: There are those who have the clinical signs although we really want to be sure because any condition can act like malaria. And since malaria is really prevalent in this area we really want it to be confirmed. And for people who insist that they have been in an area where they have not been sleeping in a mosquito net, for example, if they moved to the village and it was a bushy place, and if they also tell us that there were so many mosquitoes and it has been two or more weeks that have passed, we just give them first-line ACTs.

Interviewer: So, in such cases, you give them a first-line ACT? And what is that?

Respondent: Artemisinin-lumefantrine.

Interviewer: All right. That is quite interesting. This issue of not being able to afford a full dose clearly does not exist in this place, or you just haven't taken note of it?

Respondent: Not really. They may not afford second-line options because second-line options come with the benefits of fewer pills but usually, people want things they swallow fewer times in a day because with that, they are more adherent to the treatment than many times in a day. So, the options that I have found that usually give people a hard time are the second-line options. Also, there are different brands that are expensive. So, they can take a less-priced brand they do not like other than the other good brands that they would've liked.

Interviewer: Yeah, but they can still afford the lower price antimalarials.

Respondent: They can afford the lower-priced ones, but they cannot afford the novel drugs.

Interviewer: I think this has been quite an interesting one because when someone does not or may not afford what they want they can still be able to afford the options that you give, which is good.

Respondent: Yeah.

Interviewer: When you look at the ACTs that you give in this facility what would be the price of the cheapest ACT?

Respondent: The cheapest ACT goes for USh 5,000/=

Interviewer: And that is?

Respondent: Artefan.

Interviewer: Does it have a green leaf. Can you confirm it is okay? You can go and confirm.

Respondent: Lumartem used to have a green leaf.

Interviewer: So Artefan is the cheapest and it doesn't have a green leaf. So, which is the cheapest green leaf?

Respondent: We used to have Lumartem. Lumartem used to have a green leaf, but we no

longer have it because somehow it got lost but the green leaf Lumartem used to be USh 5,000/=

Interviewer: So right now, in the pharmacy here the green leaf that you have is sold more expensively than the Artefan?

Respondent: Yes.

Interviewer: So, the Artefan you have; one has a green leaf, and one does not have a green leaf?

Respondent: I wish we had something with a green leaf of this dosing pattern and then we compare but we don't it. We used to have a green leaf Lumartem. It was of the same dosing pattern as this one and it was the same price of USh 5,000/=

Interviewer: So, the green leaf ACT is the Artefan brand? The one that has the green leaf and the one that doesn't have a green leaf is of the same price?

Respondent: It was Lumartem. The Lumartem brand that had a green leaf is USh 5,000/= and this one is also USh 5,000/=

Interviewer: That's interesting. You previously said that you didn't consider the green leaf. It had never crossed your mind.

Respondent: I think there is just so much information and I've somehow failed to keep up. I really haven't been paying attention to the green leaf so from this time on I will pay attention.

Interviewer: So why would you sell Lumartem with a green leaf at USh 5,000/= and then Lumartem without a green leaf at USh 5,000/=?

Respondent: I think the determinant is the price we get them at. So, the price from the supplier determines the selling price. Actually, that Lumartem was actually more expensive than this Lumartem. I think this one we were getting it at around USh3,000/= and the other one we were getting it at about USh 3,600/=. So, I think you can see how the prices are different.

Interviewer: That is interesting. So, you buy the green leaf Lumartem and the non-green leaf Lumartem at different prices with the green leaf Lumartem being more expensive?

Respondent: Yeah. Lumartem was a bit expensive.

Interviewer: That is interesting. So, in terms of comparability of the prices of green leaf and the non-green leaf. There is no difference in the prices?

Respondent: No.

Interviewer: That is quite a bit of information there. We're coming to the end of the discussion though. We have talked about the ACTs and the non-ACTs. In the introduction is when we talked about the antimalarial mix. Can we follow up a little bit more on the non-ACTs that you have in this pharmacy?

Respondent: I think the only non-ACT I have is Malanil and Fancidar. We usually stock the artemisinin-combinations but for quinine, we no longer stock it. Maybe the injectables.

Interviewer: You stock the injectables?

Respondent: We stock the injections and not the tablets. And for the injections, we also have the artemisinin-based ones.

Interviewer: Do you have clients who come in to ask for non-ACTs in this facility?

Respondent: Yeah. Some people come for quinine, and some ask for chloroquine, but we do not dispense it for malaria. We usually educate the patient. There are some old conservative patients who say that it works for them. So, we have to re-educate them.

Interviewer: What is the frequency of the occurrence of such cases?

Respondent: It is a once-in-a-while thing.

Interviewer: So, when patients come to request for these non-ACTs the most commonly asked for is chloroquine?

Respondent: It is quinine.

Interviewer: And when they come to ask for quinine do they come with a prescription?

Respondent: No.

Interviewer: Is it over the counter?

Respondent: Yeah, but we do not have it. But we also tell them how the treatment guideline is. We usually do education for the patients, but some patients are stubborn; they decide to go and get it from somewhere else but even when they go, they know how

they are supposed to take the medicine.

Interviewer: So, in other words, you do not encounter prescriptions that contain non-ACTs?

Respondent: I have not really seen such a prescription here but maybe the quinine injection. But as for the oral quinine, no. There is one that I got for chloroquine for malaria treatment just once.

Interviewer: In other words, although it is not common, once in a while prescribers are prescribing non-ACTs for patients?

Respondent: Yes.

Interviewer: Why do you think they do that?

Respondent: Well, we mostly get the prescriptions from the regional referral hospital like when someone has been admitted with complicated malaria and they get the IVs, and they discharge them on orals or non-ACTs.

Interviewer: Alright. It has been an interesting interview. Is there any other issue regarding the use of ACTs or antimalarials in general in the country that you would want to share with the research team now that you have this experience?

Respondent: Yes. With the discussion, of course, I think we need more enlightenment mostly because the practice here may not be the practice out there because there are people who will walk in and be like they want Coartem and the dispenser can pick it and hand it over without educating the patient without encouraging the patient to do a quick test or something. And then about the green leaf and non-green leaf, more information should be provided to us who are in contact with the community such that we are aware that this is there, and we can benefit from the green leaf or non-green leaf and things like that.

Interviewer: Thank you so much. We will now end the interview. Thank you for your time.

Respondent: You're welcome. Thank you.

Participant - Tororo district

**CATEGORY OF INTERVIEW**	KII_PHARMACIST
**ORGANIZATION**	DRUG SHOP
**GENDER**	MALE
**DURATION OF INTERVIEW**	37:11

Interviewer: I am a research assistant-Makerere university. We are a doing a research study with a topic, predictors of local emergency and spread of artemisinin resistance among Ugandan plasmodium falciparum parasites. I am interviewing?

Respondent: (he mentions his name and pharmacy name) working with one year experience

Interviewer: Thank you sir. Have you ever heard of the word, copayment mechanism in malaria treatment?

Respondent: No

Interviewer: It is okay. Briefly, what antimalarial agents do you stock in this pharmacy?

Respondent: We have lumefantrine ACT, artemether lumefantrine, D-Artepp, quinine tablets, artesunate injection, duocotexin, P-Alaxin. That is all

Interviewer: How about the injectable antimalarial agents?

Respondent: We have artesunate and quinine injections

Interviewer: What antimalarial mix do you do here when giving to the patients?

Respondent: Actually at the pharmacy, those that we give as combinations are already combined at the factory during the manufacture, so they come when already combined. May be in the clinics where they give IVS, that is where they can combine, but here we give whole doses of tablets

Interviewer: Thank you, what determines your stocking?

Respondent: We base on the consumption data, when we see that our consumption has gone high, we depend on that?

Interviewer: Do you depend on consumption only?

Respondent: Majorly, when we are predicting out of stock, we see that some medicines are out of stock, we also depend on that

Interviewer: Have you ever heard of the word Greenleaf before as a pharmacy technician?

Respondent: Yes

Interviewer: Could you please explain to me what it means?

Respondent: Greenleaf refers to artemether-base combination therapy, they are antimalarial drugs that are derived from artemether plant.

Interviewer: Do you mean that all artemether plants qualify to be called Greenleaf?

Respondent: Most of them

Interviewer: Give me two examples of Greenleaf drugs you know?

Respondent: We have lumefantrine which is known as ACT, D-Artepp, duocotexin, P-alaxin.

Interviewer: Thank you so much, you said consumption is the major factor determining stocking here, when you stock the antimalarial drugs here, what quality control do you normally do to ensure quality is maintained?

Respondent: One, when we are stocking, the first procedure is to compare the batch numbers and the expiry date because the chances of you getting expired drugs is high. Then we look at where we are going to store our drugs, we have a temperature monitoring device to ensure that the room is suitable for storage of our drugs so that the temperature doesn't don't interfere with the drugs.

Interviewer: Apart from temperature, is there any other thing that you do?

Respondent: A lot, but because of time, you cannot deliver everything at the moment.

Interviewer: Alright, so, once you do that, do you think these products will be well stocked?

Respondent: Yes, when we stock these products and store them in conditions that are favorable and we don't have any contaminants within, we do believe our drugs are within the pharmaceutical conditions usuitable for use

Interviewer: So which procedure do you normally use when removing your drugs from store to bring them to the shelves?

Respondent: After receiving our drugs, we do control, we compare what is on the invoice then the physical stock that has been brought, after that, we go into the store. When we are removing them from the store to the shelves, we have cards we do follow, whatever item is removed from the store, we record, that is considered to be sold

Interviewer: Thank you so much, is there any other thing you would like to share with us about stocking?

Respondent: Maybe what I can add on is, sometimes it is challenging to record especially with the staff. You find that sometimes someone may forget to record when we have many customers and we are in a rush. Then I can advise is, stock cards or stock item should be provided per item whereby every item picked from the store, someone can just note down on that stock cards

Interviewer: What is the relevance of having stock cards in your facility?

Respondent: It gives you information of how much you still have at hand and how much has been given out. Then it also gives accountability of the sales and how to manage then you will be able to maintain the stock and sales

Interviewer: Thank you so much, how do clients access antimalarial drugs in this facility?

Respondent: There are 2 ways. One is under prescription, at times they may go to public facilities, they come with prescription books, due to discrepancies, you may find that the public facilities are missing out on that item and are referred to the private sectors. So, they come with their books, then we dispense the drugs with the conditions under which they have to take it. Then the second way is the client comes complaining of all the symptoms and you know this is malaria, then you can write down the prescription for that person and then give the treatment

Interviewer: Do you have all the time to write down the prescription for the patient or you just look at the signs and symptoms and treat the malaria?

Respondent: At times, a patient may mislead you when you don't tell all the information or tell you only overlying information, so when you see that the information is controversial, we may recommend that person to go to the clinic to carry out malaria test, when he confirms, he comes back with the results and there are those who don't turn up after the clinic. However, for those whom we confirm that it is malaria, we can manage

Interviewer: What are the likely signs and symptoms of malaria you would consider to give out drugs over the counter?

Respondent: One is general body pain and weakness, headache then abdominal complaints, chills. Those are the signs

Interviewer: If someone comes with a prescription with no signs and symptoms that you think should be available, what would you do?

Respondent: If such person comes, I would rather tell that person to go do a confirmation test because if I just give out the drugs, I would be causing a challenge to that person's body. So, probably, I would not give out the drugs to them

Interviewer: What really guides you to choose a given antimalarial agent to a patient that comes with no prescription but rather signs and symptoms of malaria?

Respondent: A number of factors we look at, one, patient's age, second is the other antimalarial drugs this person has used before, we also take into consideration of the severity of the malaria. However, age is a major factor because it will determine the dose and then the antimalarial drugs he/she has ever used is also important, because if that person has been using a given antimalarial for a long time, then giving him/her the same antimalarial may not bring the best results

Interviewer: Thank you so much, what is the ACT antimalarial agent purchasing behavior of clients in this facility?

Respondent: There are 2 ways. Some buy half dose. It is challenging as a few can buy a full dose. Most of them come for half or quarter dose, and when you advise them, they insist and tell you that that's what they can afford.

Interviewer: What is the approximated percentage of those who can afford a full dose treatment?

Respondent: On a scale of 10, only 4 is taking a full dose, 3 take half a dose and the rest are buying quarter

Interviewer: So you mean most of them cannot afford a full dose?

Respondent: Yes, a few can buy a full dose

Interviewer: What is the percentage of those who can afford a full dose?

Respondent: I have said only 4 out of 10 can afford a full dose

Interviewer: What do you do for the patients who cannot afford a full dose treatment?

Respondent: As a technician, I have a feeling to the treat someone to the best because reputation has to be maintained, when you treat someone to the best, you would have maintained the reputation. So, I always advise them to come back for the remaining dose because you may want to give someone a full dose because of sympathy but the fact that we buy the drugs, you put everything into consideration because of the business.

Interviewer: Is there any other thing you can do for them?

Respondent: Pardon

Interviewer: If someone comes and insists that they totally cannot a full dose, how would you address this challenge?

Respondent: I have faced this challenge before, this is common among teenagers who have given birth before finishing school, they come complaining with such and cannot buy, normally, I give them a quarter dose and recommend them to go the public facility for better treatment

Interviewer: What really determines for you to tell them to go to the public facility?

Respondent: In private facilities, we always use personal income to buy the stock, so if you continue giving out fee medicine, in a week you can get like 4 people, if you continue losing 3500 per dose times 4, that would be around 14000, that is stock gone. When I give away a quarter a dose, I know there is a certain product that can recover what I have given out. So, when I refer that person, I am really looking at saving my business. So that is what I base on to refer the patients to the public facility, because there, I am sure it is our taxes on which they are going to be treated

Interviewer: Thank you so much. What are the prices for the ACT drugs that you have?

Respondent: They vary, the lowest price is 3500, which is lonart, it can range from 6000 to 7000, then P-Alaxin tablet is 12000 to 13000

Interviewer: What is the price of the full dose of p-alaxin?

Respondent: Yes, it is what I am talking about, a full dose of 9 tablets ranges from 12000 to 13000, the syrup for infants is still the same price, duocotexin is 14000-15000, lonart DS, is 14000

Interviewer: Are those the ACTS that you have and their prices?

Respondent: Yes

Interviewer: What determines the prices of the drugs?

Respondent: One, the cost price is a key factor, then, patient capability

Interviewer: Thank you so much, pardon me on which one is the cheapest and the most expensive?

Respondent: The cheapest is ACT branded as artfan combiat, which is 3500, the most expensive is doucotexin and lonart DS which is 14000 to 15000

Interviewer: What is the difference between ACT and non-ACT, and in case, give me some examples?

Respondent: The difference starts with the first letter, ACTS are derived from artemether plants, then all the antimalarial drugs that are not derived from that plant are considered non-ACT, for instance quinine

Interviewer: Which non-ACT do you have currently in your facility?

Respondent: Currently we don't have rather than quinine

Interviewer: Thank you, why do you think a patient would ask for a non-ACT rather than an ACT?

Respondent: It is because they have been on these ACTS for some time and they are seeing recovery with ACTS being slow, so, they ask for something that gives a quick recovery.

Interviewer: How often do they come for the non-ACTS in your facility?

Respondent: It is more prescriptions, an individual coming for the non-ACT over the counter is rare. We as well rarely prescribe quinine as first treatment unless in certain conditions like pregnant mothers who is still in her first trimester or when a client has used an ACT and has not responded well, then we can prescribe quinine

Interviewer: What is the approximated percentage of patients who come for quinine?

Respondent: The percentage is very low, actually in a month, we may get no patient, so if we ever receive any, they may be 2 and even when stocking, we stocking less quinine.

Interviewer: Why do you think a patient may come for a non-ACT?

Respondent: It is because he or she is not responding to the first line he was given and is still feeling body weakness, so, he decides to come to the medical personnel so that he can change the medication, so they resort to the non-ACT

Interviewer: Thank you so much. Is there any other issue you would want to share with us?

Respondent: Yes, one, there is abuse of ACT to a big percentage. So, anyone that feels any weakness, he will go and buy ACT. That abuse has contributed to the resistance. Whenever someone feels any fever, they assume that it is malaria, so, if there is any way they can regulate so that these ACTS can be given under prescription. Then the other point is, not considering these strong drugs to be first line because in the field, I have seen people want fast recovery. Thirdly, I have observed some manufacturers prescribe for the patients, they give some money because they want to market their products, so that one as well has contributed to resistance amongst the population. So, there should be a protocol, if any

manufacturing company is to promote its product among Ugandans that they should observe. If a product is to be promoted in the population, the government should look at the consistency of the supply because the product may be effective and of high importance but will it be accessed by everyone, will it always be available, how will clients access it! Then the price should be fair, they normally sell their products expensively. That is the role of the government. The point I am trying to bring out is the manufacturers of the strong antimalarial drugs should make partnership with the government such that their products which are of great importance can be affordable and those drugs have to be supplied to the public facilities so that any person can access them.

Interviewer: Thank you so much. When you say that those products should be supplied to the public facilities, you who is running a private facility, aren't you worried that clients will run to the public facility?

Respondent: No, that is not the case because I know the population is always growing high every day, the more these clients get to know that product, the more we can sell it. In addition, there are some people who don't trust government products and also cannot handle the long lines at the public facility so they go to the private facilities. My point is they should have information and get to know about the product, that is all we need, once they know that there is type of drug, then they will go looking for it. Because the population in Uganda is high, it is challenging to the government sine it is a long process for it to deliver the medicines yet in the private sector, it is an easy process. During that process, there will be some stock outs because seasons do change and the prevalence may shoot so high yet for them they had few drugs. So, that one is not a worry.

Interviewer: What have you done on the issue of abuse of the ACTS as an individual?

Respondent I have tried to health educate these people. I have always fought with mothers who have breastfeeding infants, they always come asking for P-Alaxin syrup and when you inquire if they have ever tried some different drug, they say no. I have always tried to health educate some of them, those who can listen have taken the information serious, however, convincing some of them is not a one day process.

Interviewer: Thank you so much for the information you have shared with us. And indeed continue educating people more about antimalarial drugs and their proper use. Thank you for the interview, have a good day.

Respondent: Thank you

Participant 2 – Tororo district

**CATEGORY OF INTERVIEW**	KII_PHARMACIST
**ORGANIZATION**	DRUG SHOP
**TITLE**	PHARMACIST
**GENDER**	MALE
**DURATION OF INTERVIEW**	21:51

Interviewer: Thank you so much for accepting to undertake this short interaction on ACT purchasing behavior, stocking and pricing in a pharmacy in Tororo district. I am here with the in-charge of the pharmacy, a pharmacist. We are going to have about 7 questions, the first question is the concept of malaria copayment mechanism, have you heard about it?

Respondent: Malaria copayment, no

Interviewer: Have you encountered ACTS that have a Greenleaf label on them?

Respondent: I have not observed that.

Interviewer: There are ACTS called Greenleaf ACTS.

Respondent: No

Interviewer: So, in this pharmacy, do you stock ACTS that have a Greenleaf label on them?

Respondent: I need to stock the stock first

(they seem to be checking the medicines)

Interviewer: Had you noticed that?

Respondent: No, I had not noticed that

Interviewer: Okay, what do you think that label means?

Respondent: I see a leaf and a note there, meaning that, possibly, it clarifies where the medicine is gotten from

Interviewer: Do you find these ACTS different from ACTS that don't have Greenleaf?

Respondent: No

Interviewer: So in terms of their sale in the pharmacy, are they sold at the same price as those that don't have Greenleaf?

Respondent: As long it is artemether mefantrine which is not double strength, we usually sell them at the same price.

Interviewer: Do you have an ACT called Lonart here?

Respondent: Yes we do have lonart

Interviewer: So you sell lonart at the same price as combiat?

Respondent: Not really

Interviewer: Why is the price different?

Respondent: The purchase from the wholesalers is quite different and so also the price changes.

Interviewer: Why do you think the purchase price is different?

Respondent: Lonart is a brand that is imported, so, according to the cost of production, according to very many factors including verification fee, the wholesaler will decide to put it at a certain price, including the operational costs of the wholesaler.

Interviewer: Are you aware of antimalarials made in Uganda?

Respondent: Yes

Interviewer: What are some of them?

Respondent: There is lumartem and cedat

Interviewer: Is made by?

Respondent: Sippler

Interviewer: In this pharmacy, what range of anti-malarial drugs do you stock? If you could just identify some of them?

Respondent: We try as much as we can to follow the guidelines of the country, so we have the first line drugs which is the artemether lumefantrine, the first line alternative is artesunate and amodiaquine, that is for the uncomplicated cases. Then we have the second line which include, the most common is doucotecxin, then we have injectables, we have artesunate of varying strengths and artemether, then quinine.

Interviewer: What determines the antimalarial drugs that you stock in this pharmacy?

Respondent: One is the guidelines, because after investigations, they come up with the type of combinations that are supposed to be used depending on the resistance patterns for the country.

Interviewer: You said you had not noticed that there are some antimalarial drugs with a Greenleaf?

Respondent: Yes

Interviewer: You said you don't know what a Greenleaf means?

Respondent: Yes

Interviewer: In other words when you are stocking antimalarial drugs in particular, you had not taken note of the Greenleaf?

Respondent: No

Interviewer: You are stocking irrespective of the Greenleaf?

Respondent: Exactly, as long as we are following the guidelines.

Interviewer: In terms of patients who come to buy medicines here, could you talk to me about their purchasing behavior?

Respondent: Most of the patients, we have a divided category of antimalarials for them as majority want to purchase over the counter for any kind of combination as long as they feel feverish. And then we have the category that will come to you after testing and also those that are sent with the results from the hospitals just to come purchase that antimalarial that was not maybe available at that time.

Interviewer: Is there a situation where the dispenser is involved in deciding for the patient what antimalarials they should take?

Respondent: There are some scenarios especially when there are results, for-example when a patient comes with laboratory results. Some of them go and do the test when they feel fever, so with experience, depending on the number of parasites, you can help the client to decide but without laboratory report, no one bothers telling anything

Interviewer: From your experience, have you encountered patients who come to buy medicines from the pharmacy and price is a consideration to the patient? And how has this influenced?

Respondent: Yes, price will always be a factor when it comes to selection of medicines and we don't just give an alternative, unless, if we have evidence. Sometimes a client will be given a second line alternative usally at a higher price than the first line, so we have these debts with the patients to step them down to the first alternative, some of them will accept the clinical judgment and will adhere while others decline and end up purchasing from somewhere else

Interviewer: Are there situations where a patient cannot afford a full dose?

Respondent: Yes

Interviewer: And how have handled that in this pharmacy?

Respondent: One, we don't accept to cut any antimalarial doses for adults. As long as you are an adult, it is so uncommon unless it is for personal considerations.

Interviewer: By uncommon, you are referring to not cutting the dose?

Respondent: We only sell full doe because the moment you cut, the person will not come back and that dose is gone and that is not good for clinical practice.

Interviewer: What do you mean by "that dose is gone?"

Respondent: For-example, a client comes in and asks for 4 tablets, that is 20 left, so supposing you have a patient coming in with laboratory results and is supposed to take 3 tablets, as it is weight based, twice daily, that means you have to get 18 of those and you remain with 2 tablets, that is an illustration.

Interviewer: Are you saying that if the packaging of these tablets into tins, it would be easier to dispense them?

Respondent: Not really, right it is much easier and it ensures people take the right doses

Interviewer: How common is this occurrence where a client cannot afford a full dose? How often do you encounter this in a day?

Respondent: I cannot quantify but they are many

Interviewer: In terms of prices, what are the different prices for the ACTS that you have stocked in this facility?

Respondent: Duocotexin is 15000- full dose of 9 tablets, then we have alternative which are close to duocotexin which is 10000, redmol, P-Alaxin, then artmether lumefantrine lonart for adults ranges from 10000 to 12000, then we lonart for the pediatrics which is the same price as lonart, the other lonart range from 3000-3500, then injectables they range from 4500 and above

Interviewer: Thank you, what is the cheapest dispensing price for artmether lumefantrine,?

Respondent: 3500

Interviewer: What is the most expensive price for artemether lumefantrine?

Respondent: 3500

Interviewer: For artemether lumefantrine?

Respondent: Any brand, that is 12000 and that is lonart

Interviewer: And it is not Greenleaf. Right?

(silence, they check the boxes)

Interviewer: Lonart doesn't have Greenleaf and it is the most expensive, and the one that is the cheapest has Greenleaf and it is 3500. What are the determinants of the dispensing prices?

Respondent: One is price we buy it, but for artemether lumefantrine, prices are constant at 3500, what may change the price may be the cost

Interviewer: And the constant 3500, you had not paid attention to see that it could be the Greenleaf or you have another reason why the price is 3500?

Respondent: Yes the Greenleaf may also come into consideration because it is something that we have actually paid attention to, but also the cost influences most.

Interviewer: In terms of the artemisinins, which are the first line, there also non-artemisinins, do you stock them in this pharmacy?

Respondent: Yes, we do

Interviewer: What are the artemisinins that you stock in this pharmacy?

Respondent: Quinine is one of them, erythromycin especially to pregnan women who cannot adhere to the rest of the antimalarial drugs, then we have the common fansidar.

Interviewer: How often do you encounter patients who come to request for non-ACTS?

Respondent: It is common

Interviewer: And what is the most commonly requested for?

Respondent: Fansidar, mostly by the sicklers and pregnant mothers but rarely by pregnant mothers because most of them are covered

Interviewer: And when they come to buy these artemisinins, do they usually have prescriptions or not?

Respondent: Yes, some of the clients we dispense to medicine, we make sure they have prescriptions, the ones we dispense to without prescription are regular clients

Interviewer: Thank you so much, we have come to the end of this discussion, is there any other thing you would like to bring to our attention regarding the area of discussion so far?

Respondent: All I can say is I am so happy to have given my input and I am so happy that such programs and such a trial of research is being conducted within the country because it is enforcing good clinical practice and protection of the few antimalarial drugs that were discovered. Also, ensure you complete the study and let us know when the data has been analysed and processed and ready.

Interviewer: We will try to make sure that the report of the study is availed to all the stakeholders, the ministry and the different stakeholders at the study sites. Also this will be published, we have a report website and the results will be available on the website. Thank you very much
