# Supplementary material for: Experience of healthcare personnel on Co-payment mechanism and the implications on its use in private drug outlets in Uganda
Source: PLoS One. 2024 May 17;19(5):e0297416. doi: 10.1371/journal.pone.0297416 (PMC11101086; doi:10.1371/journal.pone.0297416)
Supplement: Attachment — Submitted filename: Response to reviewers_Experiences.docx [file pone.0297416.s003.docx]

**Response to reviewers comments for manuscript PONE-D-24-00298**

Thanks for the comments on our manuscript, responding to the comments have helped further improve the manuscript.

**Requirment comment #1:** 1. Please ensure that your manuscript meets PLOS ONE's style requirements, including those for file naming. The PLOS ONE style templates can be found at

<https://journals.plos.org/plosone/s/file?id=wjVg/PLOSOne_formatting_sample_main_body.pdf> and 
<https://journals.plos.org/plosone/s/file?id=ba62/PLOSOne_formatting_sample_title_authors_affiliations.pdf>

**Response:** Thanks this has been rectified in the revised manuscritp.

**Requirment comment #2:**  In the Methods, please provide detailed information about the procedure by which informed consent was obtained from organ/tissue donors or their next of kin. In addition, please provide a blank example of the form used to obtain consent from donors, and an English translation if the original is in a different language.

**Response:** Thanks for the comment, the statement on procedure for obtaining informed consent has been provided in the revised manuscritp. The blank form used for obtaining the consent has also been provided in the revised manuscript.

**Requirment comment #3:**  Thank you for stating the following financial disclosure: 
"This study is part of the EDCTP2 programme supported by the European Union (TMA2019CDF-2662- Pfkelch13 emergence)."

Please state what role the funders took in the study.  If the funders had no role, please state: ""The funders had no role in study design, data collection and analysis, decision to publish, or preparation of the manuscript."" If this statement is not correct you must amend it as needed.

Please include this amended Role of Funder statement in your cover letter; we will change the online submission form on your behalf.

**Response:** The statement, "The funders had no role in study design, data collection and analysis, decision to publish, or preparation of the manuscript.” Is correct and has been provided as guided.

**Requirment comment #3:**  We note that your Data Availability Statement is currently as follows: "All relevant data are within the manuscript and its Supporting Information files."

Please confirm at this time whether or not your submission contains all raw data required to replicate the results of your study. Authors must share the “minimal data set” for their submission. PLOS defines the minimal data set to consist of the data required to replicate all study findings reported in the article, as well as related metadata and methods (<https://journals.plos.org/plosone/s/data-availability#loc-minimal-data-set-definition>).

For example, authors should submit the following data:

- The values behind the means, standard deviations and other measures reported;
- The values used to build graphs;
- The points extracted from images for analysis.

Authors do not need to submit their entire data set if only a portion of the data was used in the reported study.

If your submission does not contain these data, please either upload them as Supporting Information files or deposit them to a stable, public repository and provide us with the relevant URLs, DOIs, or accession numbers. For a list of recommended repositories, please see <https://journals.plos.org/plosone/s/recommended-repositories>.

If there are ethical or legal restrictions on sharing a de-identified data set, please explain them in detail (e.g., data contain potentially sensitive information, data are owned by a third-party organization, etc.) and who has imposed them (e.g., an ethics committee). Please also provide contact information for a data access committee, ethics committee, or other institutional body to which data requests may be sent. If data are owned by a third party, please indicate how others may request data access.

**Response:** Thanks for the comment, this being a qualitative study we have audio recordings which were analyzed to come up with the current results. The ethics do not allow for sharing of the audio recordings of individual interviews. We have however, provided the interview guide that was used in conducting the interviews in the revised manuscript.

**Requirement comment #5:** When completing the data availability statement of the submission form, you indicated that you will make your data available on acceptance. We strongly recommend all authors decide on a data sharing plan before acceptance, as the process can be lengthy and hold up publication timelines. Please note that, though access restrictions are acceptable now, your entire data will need to be made freely accessible if your manuscript is accepted for publication. This policy applies to all data except where public deposition would breach compliance with the protocol approved by your research ethics board. If you are unable to adhere to our open data policy, please kindly revise your statement to explain your reasoning and we will seek the editor's input on an exemption. Please be assured that, once you have provided your new statement, the assessment of your exemption will not hold up the peer review process.

**Response:** This was a qualitative study where we audio recorded the respondents and analyzed the audio interviews for this manuscript. A sample of the approved interview guide which was used in the interviews is provided in the revised manuscript.

**Requirement comment #6**. Please include your full ethics statement in the ‘Methods’ section of your manuscript file. In your statement, please include the full name of the IRB or ethics committee who approved or waived your study, as well as whether or not you obtained informed written or verbal consent. If consent was waived for your study, please include this information in your statement as well.

**Response:** A full ethics statement has been provided in the revised manuscript as guided.

5. Review Comments to the Author

Please use the space provided to explain your answers to the questions above. You may also include additional comments for the author, including concerns about dual publication, research ethics, or publication ethics. (Please upload your review as an attachment if it exceeds 20,000 characters)

Reviewer #1: This study conducted key informant interviews with 25 pharmacists/dispensers in two districts in Uganda to better understand types of anti-malarials stocked and sold, as well as clients’ purchasing behaviors. This study was done in the context of the Global Fund’s Co-payment mechanism which seeks to lower the cost of quality-assured ACTs for first-line buyers of the drug to improve access, particularly given that many people seek care for malaria in the private sector. The study found that most people purchase anti-malarial drugs without a prescription and that many people buy less than a complete dose of ACTs. While the latter is a particularly interesting result, I think the authors need to connect their conclusions more clearly to the evidence to make it more convincing.

**Response:** This has been rectified in the revised manuscript.

Comments:
**Reviewer #1 comment 1:** I would have liked more description of the sampling strategy. How were districts chosen? How were high/low malaria transmission defined?

**Response:** We used the Ministry of health stratification of malaria transmission intensity across the country to identify areas categorized as having moderate-to-high and low malaria transmission. Malaria transmission in the country is stratified into four levels based on malaria cases, ‘high’ (> 499 cases per 1000 population/year), ‘moderate’ (200 to 499 cases per 1000 population per year), ‘low’ level 2 (50 to 199 cases per 1000 population per year), ‘very low’ level 1 (1-49 cases per 1000 population per year) (Zalwango et al., 2024). Tororo and Apac are districts with moderate-to-high malaria transmission while Mbarara and Kabale are low malaria transmission area (Nankabirwa et al., 2022; Zalwango et al., 2024). The districts were purposely selected following malaria transmission intensity, moderate-to-high (Apac and Tororo) and low (Mbarara and Kabale).

Nankabirwa JI, Bousema T, Blanken SL, Rek J, Arinaitwe E, Greenhouse B, et al. Measures of malaria transmission, infection, and disease in an area bordering two districts with and without sustained indoor residual spraying of insecticide in Uganda. PLoS One 2022;17(12):e0279464.

Zalwango MG, Zalwango JF, Kadobera D, Bulage L, Nanziri C, Migisha R, et al. Evaluation of malaria outbreak detection methods, Uganda, 2022. Malar J. 2024;23(1):18.

**Reviewer #1 comment** 2. Was a listing of all pharmacies selling anti-malarials done in these two districts? If not, how were the pharmacies selected? How was it decided which shops to visit first?

**Response:** Thanks for the comment, In each district (Tororo, Apac, Mbarara and Kabale), a comprehensive list of the available private drug outlets was compiled using the National drug authority (NDA) register of drug outlets. Additionally, a census was taken to identify all functional private drug outlets (pharmacies and drug shops) in each district prior to data collection. From the census and review of drug outlets listed in the NDA register, each of the available drug outlet in the survey districts were considered for inclusion in the survey. All the drug outlets which reported stocking antimalarial agents were noted and healthcare personnel (Pharmacist/dispenser) contacted for enrolment into the study. In each district, drug outlets in urban settings were visited first followed by those in rural settings.

**Reviewer #1 comment 3**. I would also have liked more detail on the settings of these pharmacies and about the pharmacies itself.
a. For example, is there a big difference in socio-economic status between the two districts (that might explain why in one district people tended not to buy a complete dose)?
b. How big were the pharmacies? How many people visited them per day? Were they in an urban or rural setting?

**Response:** The study was done in four districts, Apac, Tororo, Mbarara and Kabale district. Apac and Tororo have moderate-to-high malaria transmission while Mbarara and Kabale have low malaria transmission. We didn’t explore to establish why malaria patients in some districts were able to purchase full dose while those in other districts were not able. The economic activities in all the four districts is mainly agriculture and farming. We collected data from retail pharmacies in all the four districts. In this study, we didn’t assess how many people visited each pharmacy per day. In each district, data was collected from pharmacies located in both urban and rural settings.

**Reviewer #1 comment 4**. How long did the interviews take? I suggest including the interview questions in the appendix.

**Response:** Thanks for the comment, the interview guide has been provided as supplementary information in the revised manuscript.

**Reviewer #1 comment 5**. I would move the information on refusals into the results section. Is there any information on why they might have refused?

**Response:** Thanks for the comment, we sought consent from participants prior to being interviewed. Since participation in the study was voluntary we didn’t probe for the reasons of refusal to participate in the study. The information on refusals has been also included in the results section.

**Reviewer #1 comment 6**. The themes could be more descriptive in terms of what actual results were. For example, instead of “Considerations taken while stocking, and pricing of antimalarial agents in private drug outlets”, the authors could say something like “Antimalarial stocking is influenced by price and client demand”

**Response:** Thanks for the comment, this has been rectified in the revised manuscript.

**Reviewer #1 comment 7**. Many of the results do not include supporting quotes, which make them hard to evaluate. I think the paper would be stronger if the authors include quotes (or multiple quotes) for all statements. For example:
a. Lines 213 (about the national guidelines)
b. Line 277 (about selling in installments)
c. Line 283 (about providing antimalarials on credit)
d. Line 245 (about stocking antimalarials not being a priority)

**Response:** Thanks for the comment, sorry for this ommission in the initial write up. All the supporting quotes have been incorporated in the revised manuscript.

**Reviewer #1 comment 8**. Some of the statements seem quite strong and it might be helpful to get a sense of how many pharmacists actually reported/mentioned something (a few? some? all?). The authors often say “Study participants reported” or “Pharmacists reportedly sold…” but it’s not clear how many actually said this.

**Response:** Thanks for the comment, this has been corrected throughout in the revised manuscript.

**Reviewer #1 comment 9**. The authors say that most drug client outlets who want to buy ACTs can’t afford full dose. Did all the pharmacists report this and how do they know that it’s because of the cost? I did not find the one quote that was included on this (Line 364) particularly convincing as it just mentioned a client buying a pediatric dose.

**Response:** Thanks for the comment, this was not reported by all the pharmacists. The inability to purchase full dose was common in Apac and Tororo districts areas with moerate-to-high malaria transmission compared to Kabale and Mbarara and is reported in the manuscript. The associated quotes are also provided in the manuscript.

**Reviewer #1 comment 10**. In the abstract it is mentioned that 7/10 clients come without a prescription. Where does this number come from? Did the pharamacists report this?

**Response:** Thanks for the comment, this was reported by the pharmacists during the interview as a rough estimate of the clients who come to purchase ACTs in the pharmacies without a prescritpion on a daily basis. This has been corrected in the revised manuscript.

**Reviewer #1 comment 11**. Do the authors have information on the cost of ACTs across the different pharmacies? It might be helpful to report the range of prices for the main types of anti-malarials to verify that ACTs do, in fact, cost more than the other anti-malarials despite the co-payment mechanism.

**Response:** This was assessed and reported in a quantitative study that we did seperately and the manuscript is under review. However, for the qualitative study the focus was mainly on the experiences of healthcare personnel with the Copayment mechanism.

**Reviewer #1 comment 12**. Lastly, it was not clear to me why pharmacists’ knowledge of the copayment mechanism was important. Since it’s a subsidy to first-line buyers, we wouldn’t necessarily expect the pharmacists to know about it, or that it would affect their selling decisions.

**Response:** The study was done among retail pharmacies in communities. In the country there are both subsidized and non-subsidized ACTs in circulation. The pharmacists who run most of the retail pharmacies from time to time stock their pharmacies with various medicines. Their knowledge of Copayment mechanism would help guide the choice of ACTs to stock in the pharmacies. As found from our study, pharmacists reported having fewer subsidized ACTs (QAACT) in stock compared to non-subsidized ACTs with some even having non-ACT antimalarials like chloroquine whose use for malaria treatment was discontinued in the country. This could be due to lack of awareness and knowledge on Copayment mechanism. It is therefore important that the pharmacists in retail drug outlets are aware and knowledgible on Copayment mechanism to help guide stocking and dispensing of ACTs in community pharmacies in the country.

Reviewer #2: Title: Experience of healthcare personnel on Co-payment mechanism and the implications on its use in private drug outlets in Uganda.

**Reviewer #2 General comment:** A well written manuscript exploring experiences of healthcare personnel on Co-payment mechanism and the implication on its use in private drug outlets in Uganda.
There are a few comments that need to be attended:

**Response:** Thanks for the comment, addressing the comments have helped further improve the manuscript.


**Reviewer #2 comment:** Lines 91 & 92: QAACT & ACTs should be first written in full on initial appearance. Rather ACT is subsequently written in full in line 468.

**Response:** Thanks for the comment, ACT is first used and written in full in line 47 while QAACT is first used and written in full in line 57 of the revised manuscript.

**Reviewer #2 comment:** Line 243-252: text and accompanying quote do not tally.

**Response:** This has been rectified through out the revised manuscript.

**Reviewer #2 comment**: Line 346: statement......about seven in ten clients do not present prescriptions when seeking to purchase antimalarial agents... this phrase appear to present information as as though it originated from quantitative data. There is need to rephrase it.

**Response:** Thanks for the comment, this has been rectified in the revised manuscript.
