# Supplementary material for: Experience of healthcare personnel on Co-payment mechanism and the implications on its use in private drug outlets in Uganda
Source: PLoS One. 2024 May 17;19(5):e0297416. doi: 10.1371/journal.pone.0297416 (PMC11101086; doi:10.1371/journal.pone.0297416)
Supplement: Attachment — Submitted filename: Response to Reviewers_10April2024.doc [file pone.0297416.s004.doc]

**Response to Reviewer’s comments on manuscript:** PONE-D-24-00298R1

**We are grateful for the comments, addressing the comments have helped further improve the manuscript.**

**Comment #1, Reviewer #1:** Thank you for clarifying the sampling procedure and for adding the additional quotes- I believe they have considerably strengthened the paper. I have a few remaining minor comments:

**Response:** Thanks for the comment

**Comment #2, Reviewer #1:** . I still find the 7/10 number cited in the abstract and in Line 449 very specific (and potentially misleading) for a study based on qualitative interviews. Did multiple pharmacists all give this specific estimate? I suggest removing the number and just saying something like “pharmacists said that many clients don’t come with a prescription.”

**Response:** Thanks for the comment, this has been corrected all through the revised manuscript as guided to, “pharmacists said that many clients don’t come with a prescription.”

**Comment #3, Reviewer #1:**  It would be helpful to note as a limitation that the study was conducted in only 4 districts and results may be different in other areas.

**Response:** This has been noted, the limitation has been incorporated in the revised manuscript.

**Comment #4, Reviewer #1:**  Line 203: It would be good to include a citation for the software.

**Response:** Thanks, the reference for the software has been included in the revised manuscript.

**Comment #5, Reviewer #1:**  I suggest including a note for each participant quote indicating whether they come from a high vs a low transmission district. This might help contextualize the comments a bit.

**Response: Thanks for the comment, this has been included in the revised manuscript.**

**Comment #6, Reviewer #2:** I find that the authors have not adequately addressed the data availability statement issue raised in the "requirements".

**Response:** We have provided the key informant guide that was used in the interviews and a transcript from an audio recording of an interview.

**Reference**

ATLAS.ti Scientific Software Development GmbH (2021): ATLAS.ti Windows. V. 9.0.0.214 ATLAS.ti Scientific Software Development GmbH. Windows, Berlin
